# Kinetics of Nanomedicine in Tumor Spheroid as an *In Vitro* Model System for Efficient Tumor-Targeted Drug Delivery With Insights From Mathematical Models

**DOI:** 10.3389/fbioe.2021.785937

**Published:** 2021-12-01

**Authors:** Sayoni Maitra Roy, Vrinda Garg, Sourav Barman, Chitrita Ghosh, Amit Ranjan Maity, Surya K. Ghosh

**Affiliations:** ^1^ Amity Institute of Biotechnology, Amity University, Kolkata, India; ^2^ Department of Physics, National Institute of Technology, Warangal, India; ^3^ Department of Pharmacology, Burdwan Medical College and Hospital, Burdwan, India

**Keywords:** *in vitro* cell culture, multicellular tumor spheroids, tumor microenvironment, nanomedicine, tumor-targeted drug delivery, tumor penetration and accumulation, mathematical modeling

## Abstract

Numerous strategies have been developed to treat cancer conventionally. Most importantly, chemotherapy shows its huge promise as a better treatment modality over others. Nonetheless, the very complex behavior of the tumor microenvironment frequently impedes successful drug delivery to the tumor sites that further demands very urgent and effective distribution mechanisms of anticancer drugs specifically to the tumor sites. Hence, targeted drug delivery to tumor sites has become a major challenge to the scientific community for cancer therapy by assuring drug effects to selective tumor tissue and overcoming undesired toxic side effects to the normal tissues. The application of nanotechnology to the drug delivery system pays heed to the design of nanomedicine for specific cell distribution. Aiming to limit the use of traditional strategies, the adequacy of drug-loaded nanocarriers (i.e., nanomedicine) proves worthwhile. After systemic blood circulation, a typical nanomedicine follows three levels of disposition to tumor cells in order to exhibit efficient pharmacological effects induced by the drug candidates residing within it. As a result, nanomedicine propounds the assurance towards the improved bioavailability of anticancer drug candidates, increased dose responses, and enhanced targeted efficiency towards delivery and distribution of effective therapeutic concentration, limiting toxic concentration. These aspects emanate the proficiency of drug delivery mechanisms. Understanding the potential tumor targeting barriers and limiting conditions for nanomedicine extravasation, tumor penetration, and final accumulation of the anticancer drug to tumor mass, experiments with *in vivo* animal models for nanomedicine screening are a key step before it reaches clinical translation. Although the study with animals is undoubtedly valuable, it has many associated ethical issues. Moreover, individual experiments are very expensive and take a longer time to conclude. To overcome these issues, nowadays, multicellular tumor spheroids are considered a promising *in vitro* model system that proposes better replication of *in vivo* tumor properties for the future development of new therapeutics. In this review, we will discuss how tumor spheroids could be used as an *in vitro* model system to screen nanomedicine used in targeted drug delivery, aiming for better therapeutic benefits. In addition, the recent proliferation of mathematical modeling approaches gives profound insight into the underlying physical principles and produces quantitative predictions. The hierarchical tumor structure is already well decorous to be treated mathematically. To study targeted drug delivery, mathematical modeling of tumor architecture, its growth, and the concentration gradient of oxygen are the points of prime focus. Not only are the quantitative models circumscribed to the spheroid, but also the role of modeling for the nanoparticle is equally inevitable. Abundant mathematical models have been set in motion for more elaborative and meticulous designing of nanomedicine, addressing the question regarding the objective of nanoparticle delivery to increase the concentration and the augmentative exposure of the therapeutic drug molecule to the core. Thus, to diffuse the dichotomy among the chemistry involved, biological data, and the underlying physics, the mathematical models play an indispensable role in assisting the experimentalist with further evaluation by providing the admissible quantitative approach that can be validated. This review will provide an overview of the targeted drug delivery mechanism for spheroid, using nanomedicine as an advantageous tool.

## 1 Introduction

Cancer tissues are anomalous cell mass exhibiting escalated growth and unregulated cell proliferation. They divide at abnormal rates, which enable them to escape apoptosis when they ought to. For the last several decades, among various strategies (Nakamura and Harashima, 2017) used to treat intractable cancer, chemotherapy has shown its promise as a better therapeutic strategy over others. However, a complex tumor microenvironment and its hierarchical structure often impede successful drug delivery to tumor sites (Junttila and Sauvage, 2013; Frankel et al., 2017; Musetti and Huang, 2018; Wang et al., 2018; Arneth, 2019; Baghban et al., 2020). It further demands a commendable and effective delivery strategy of anticancer drug candidates, like small molecular anticancer drugs, therapeutic nucleic acid, therapeutic protein, and therapeutic peptide, specifically to the diseased site (Maity and Stepensky, 2016; Takashi et al., 2017). Furthermore, the poor water solubility of many anticancer drugs, untoward pharmacokinetics, and the related underlying risk of cytotoxic effects in normal tissue put drug candidates to use for further clinical applications (Li et al., 1986; Kusumoto et al., 1990; Onoue et al., 2014). Additionally, the limited delivery efficiency of drug candidates to selective tumor sites makes treatment efficacy remarkably poor. Hence, targeted delivery of drugs to tumor sites has become a major scientific challenge for cancer therapy by assuring drug efficacy to selectively diseased sites and overcoming undesired cytotoxic side effects to normal tissue (Iqbal et al., 2017; Srinivasarao and Low, 2017; Unsoy and Gunduz, 2018).

**FIGURE 1:**
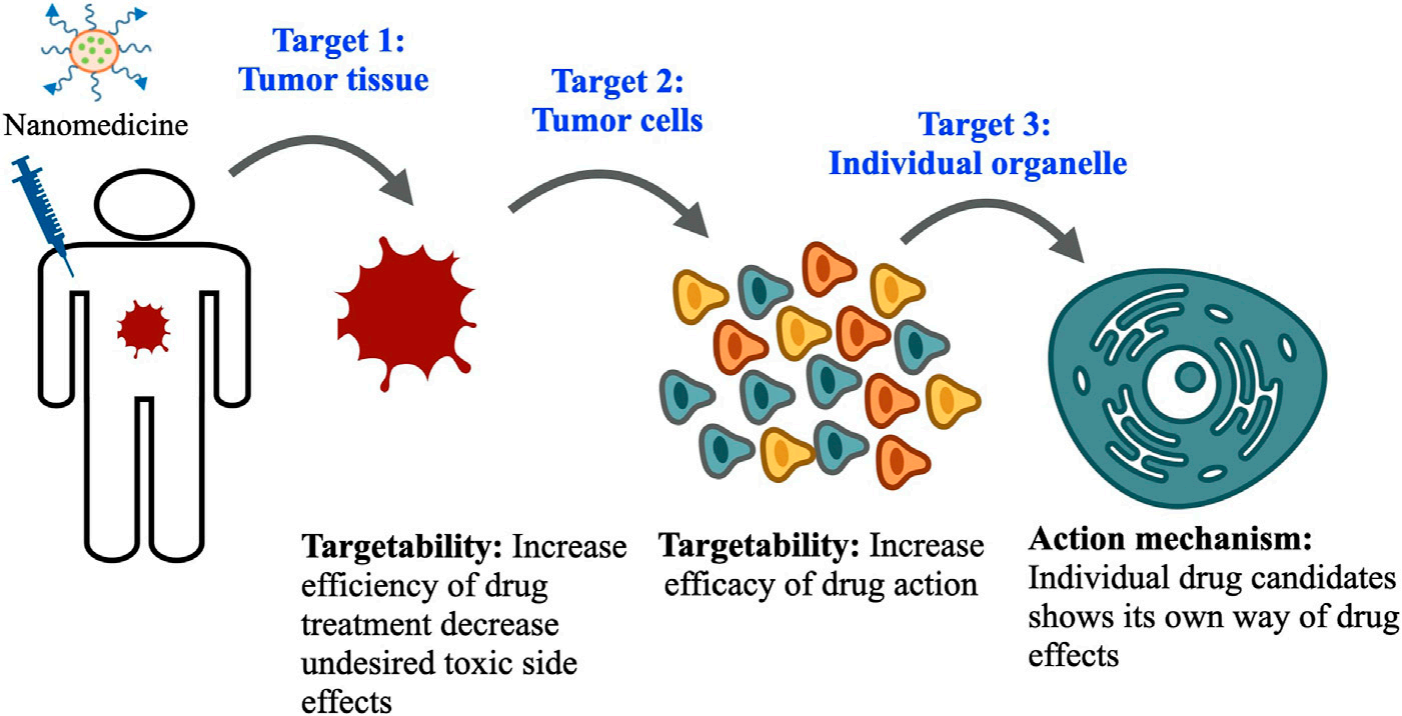
Three levels of drug disposition: The nanomedicine faces three levels of dispositions before reaching its final target. First, they enter the tumor tissue, where multiple complex factors in the tumor microenvironment limit their delivery; second, they follow cellular endocytosis mechanisms and enter inside the tumor cell and exert desired therapeutic effects. Finally, the drug candidate inside nanomedicine reaches individual organelles to exhibit its action mechanism.

Toward this, nanomedicine has already proven its indispensable part in addressing these issues. An efficient nanomedicine can circulate in the blood compartment stably for a longer period of time and get partially engulfed by macrophages of the reticuloendothelial system with extravasation to the tumor site. This successful extravasation facilitates its interaction with tumor tissue for further recognition and uptake by target cells. At the same time, nanomedicine also exhibits poor extravasation at the normal tissue region and a reasonably small amount of distribution over there due to tight and continuous vasculature. After systemic circulation of nanomedicine in blood, it follows three levels of disposition to tumor cells in order to exhibit the required pharmacological effects induced by the drug candidates residing within (Figure 1). At the first level, nanomedicine relocates itself from blood capillaries to tumor sites, which is beneficial for treatment efficiency. Subsequently, at the tumor site, nanomedicine distributes itself to each and individual tumor cell, which is highly desirable, although multiple complex factors in the tumor microenvironment resist its entry to the tumor cell. Ultimately, individual drug candidates reach subcellular organelles to perform their actions. So, nanomedicine helps improve the bioavailability of drug candidates, increase dose responses, and enhance targeting efficiency towards delivering and distribution of effective therapeutic concentration and by limiting toxic concentration. This characteristic of nanomedicine shows its promise towards improved therapeutic efficacy of anticancer drugs (Martin et al., 2017; John et al., 2020; Ferreira et al., 2021). In the preclinical study, the distribution of nanomedicine in tumor cells following its disposition in tumor tissue is the key investigation to determine the efficiency of drug treatment and subsequent disease management strategy.

Before reaching clinical translation, understanding the pharmacokinetics of the anticancer drug at different levels of drug disposition in *in vivo* animal modeling is a very important step. Hence, screening nanomedicine in animal models is essential. Although performing experiments with animal models is undoubtedly valuable, many ethical protocols should be followed to conduct an experiment with animals (Patel et al., 2015). Moreover, animal experiments are very expensive, take a longer time to finish, and require repeated experimentation on individual groups to reach the final conclusion. Cancer cells are now being grown in a controlled environment in the laboratory to reproduce the characteristics of *in vivo* solid tumors. Creating the same environment artificially as the real tumor is subject to a lot of constraints in terms of progressive cell accumulation, properties that help cancer cells persist within the tissue leading to a tumor, and physicochemical traits that result in their invasiveness and drug resistance. To overcome these issues, multicellular tumor spheroids are considered promising *in vitro* three-dimensional (3D culture) models, which are intermediate between *in vitro* cellular monolayer and *in vivo* animal models in terms of complexity, cell-cell communication, and gradients of nutrients and oxygen (Kostarelos et al., 2004; Mehta et al., 2012; Solomon et al., 2016). Thus, these *in vitro* tumor models overcome the ethical issues concerned with animal models as well. Tumor spheroids are formed artificially and very easily under laboratory conditions by aggregation and inducing self-assembly of tumor cells, representing a three-dimensional architecture, and can closely mimic drug penetration, distribution, and final accumulation in cancer cells as that of the solid tumor in the body (Sant and Johnston, 2017b; Gianpiero et al., 2017). Moreover, they can assess the efficacy of anticancer drugs as they can serve as a close representative of tumor tissue and cellular microenvironment in terms of cell proliferation, heterogeneity, and drug resistance. Tumor spheroid can replicate the tumor microenvironment where *in vivo* parameters like gradients of soluble cell culture components (e.g., oxygen, nutrients, and growth factor) and cellular waste are generated after metabolic activities (paracrine factors, different metabolites, etc.). These gradients impose a barrier for the diffusion of nanomedicine in the spheroid architecture. Moreover, spheroids build a complex cell-cell network and cell-extracellular matrix adhesions. Thus, 3D tumor spheroid models possess several characteristics like solid tumors such as cell-cell interactions, cellular microenvironments (e.g., hypoxia), drug penetration, reaction, resistance, and extracellular matrix (ECM) production and deposition. Cellular organization within the tumor spheroid is the key aspect governing impaired therapeutic efficacy of anticancer drugs. The proliferating external cell layer of the spheroid causes higher consumption of oxygen and the concentrations of oxygen and nutrients are reduced dramatically towards the center of the spheroid. This hypoxic environment at the center displays an unregulated expression of the hypoxia-inducible factor (HIF), which contributes to establishing therapeutic resistance mechanisms. Both hypoxia and necrosis play crucial roles in anticancer drug resistance mechanisms (Karsch-Bluman et al., 2019; Sharma et al., 2019; Karsch Bluman and Benny, 2020).

**FIGURE 2:**
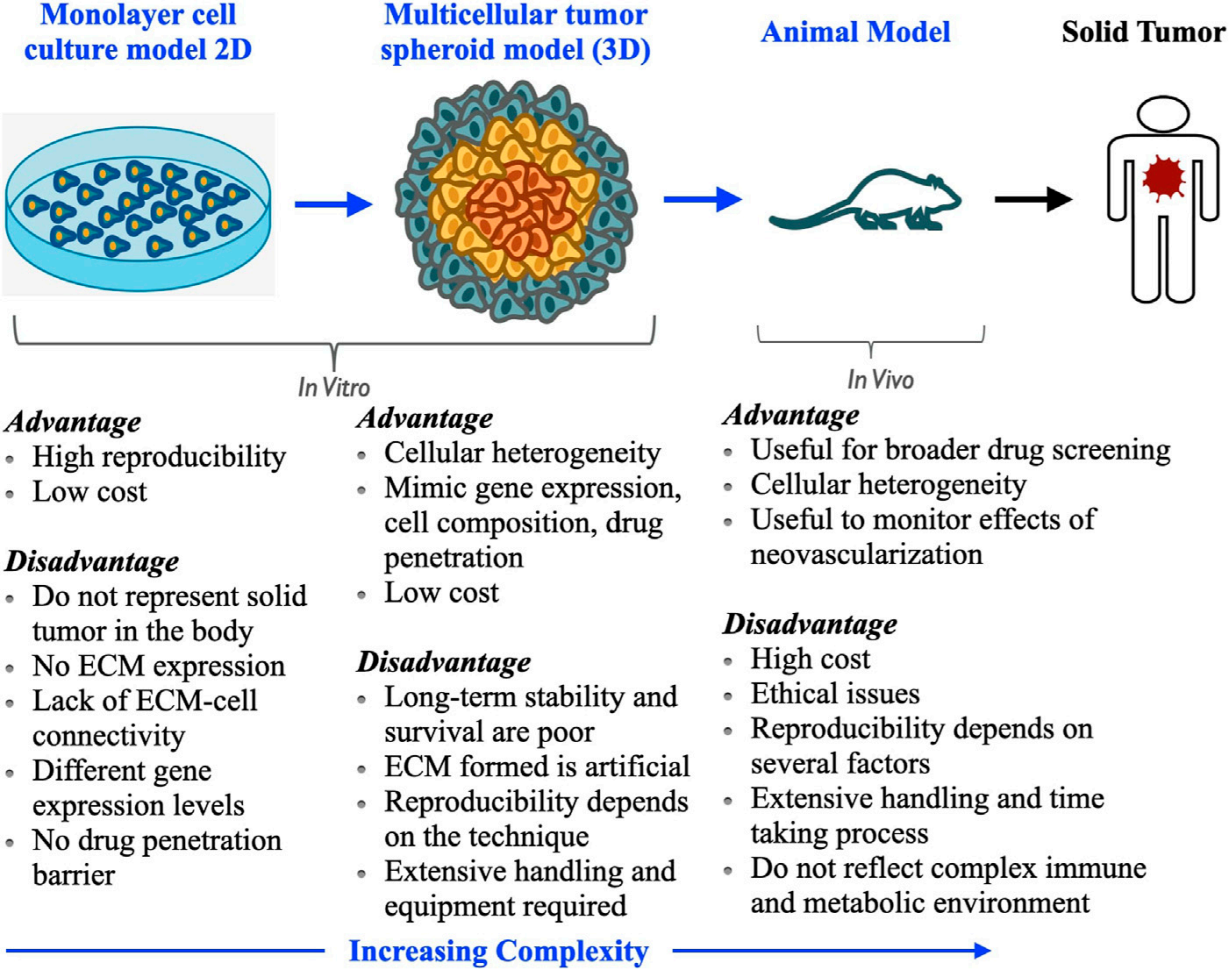
Comparison of various tumor models used for drug screening: importance of multicellular tumor spheroid as an *in vitro* model in preclinical level for screening of new anticancer drugs and development of treatment.

For several decades, traditional two-dimensional *in vitro* monolayer cell cultures (2D culture) have been used to screen therapeutics for different intractable diseases, including cancer. Matching between the intrinsic microenvironment and heterogeneity as a real solid tumor is lacking in these cell cultures. Additionally, despite their relative ease of handling, reproducibility, and affordable establishment cost, 2D monolayer cell cultures lag in cell-cell signaling, penetration profile of drugs, and their accumulation as solid tumors (Kapałczyńska et al., 2018). Thus, the therapeutic strategies and *in vitro* methodologies can be indeed improved by considering that the three-dimensional cell cultures maintain the similar complex physiology and microenvironment as a real solid tumor. The tumor spheroid bridges the gap between the 2D cultures and animal models. This model allows replicating the architecture of solid tumors and better investigates the pathobiology of human cancer (Figure 2). The potential of the tumor spheroid model is reported to be particularly needful for the development of new anticancer strategies and better measures for cancer treatment and is well acclimated for high-throughput drug screening. Currently, chemotherapy is considered one of the most promising and first-line treatment methods among different anticancer therapies; thereby, before reaching any conclusion about performing animal experiments to translate nanomedicine formulations from bench to bedside, initial screening of the tumor spheroid model is highly needed. For a more elaborative view and a thorough understanding of the mechanism, mathematical modeling can be used. It can give a direction to the new approaches for treating the system quantitatively (Goodman et al., 2008; Altrock et al., 2015). The essence of numerical modeling lies in developing the mathematical formulations representing the underlying physical mechanisms of the 3D tumor spheroid growth rate and kinetics of nanoparticles through its cellular organization (Chou et al., 2013). The quantitative approach at distinct cellular scales of tumor spheroid architecture and diffusion of drug molecules or nanodrug formulations (i.e., nanomedicine) inside a tumor and its crowded environment (Ghosh et al., 2012; Ghosh et al., 2015; Ghosh et al., 2016) provides the momentum for the optimized study of these mechanisms. These models can be validated by experimental data bringing forth the unknown parameters that can assist the qualitative approach more precisely (Hori et al., 2021).

In this article, we will discuss how tumor spheroids could be used as an *in vitro* model system to screen nanomedicine used in tumor-targeted drug delivery aiming to better therapeutic benefits of anticancer drugs for future development of new therapeutics and treatment strategies at the preclinical stage (Mu et al., 2018; Raza et al., 2018; Baker et al., 2020). Here, we will also provide an overview of qualitative description, including the strategies to approach the system quantitatively at distinct scales and coordinates integrating both solid tumor and nanomedicine as one system for tumor-targeted drug delivery (Altrock et al., 2015) Beginning with the hierarchical architecture of the tumor spheroid and then its microenvironment (Laird, 1964), we will discuss the mathematical modeling at the cellular, subcellular, and extracellular scale using molecular dynamics, reaction-diffusion mechanism, and hybrid models (Kim and Stolarska, 2007). The latter section will elucidate the physicochemical properties of nanomedicines with a quantitative description of the concentration of drug molecules, and binding sites on cell surface, along with models for adsorption, internalization (Wilhelm et al., 2002; Gao et al., 2013), and diffusion of nanomedicine through the spheroid taking intricate cell-cell interaction, cell-ECM adhesions, and porous gel matrix into consideration for the evaluation of nanomedicine penetration (Ghosh et al., 2015; Ghosh et al., 2016), featuring their adequacy as an insightful tool (Goodman et al., 2008; Ghosh et al., 2012). We conclude with an outlook of the future perspective of the dynamics of shape change of nanomedicine as a modified and efficient drug delivery system (Li et al., 2017b).

## 2 Solid Tumor

A tumor is an abnormal lump of cells showing dysregulated proliferation. Tumors can be largely divided into two categories: non-solid tumors and solid tumors. Non-solid tumors are generally referred to as those having a hematological origin. Examples include lymphoma and leukemia. On the other hand, solid tumors are structures made up of an abnormal mass of tissues, which do not contain cysts or liquid areas. They may be benign or malignant (Gavhane et al., 2011). Benign tumors are generally slow-growing, resemble normal cells, and remain localized. In contrast, malignant tumors are cancerous bearing characteristics like anaplasia, invasiveness, and metastasis. The nomenclature of solid tumors is based on the type of cells that form them. For example, osteosarcoma and neurofibrosarcoma are comprised of bone cells and nerve sheath cells, respectively (Gavhane et al., 2011). Solid tumors are heterogeneous entities in which the progression is governed by crosstalk between the epithelial parenchyma of carcinomas and the supportive framework of a tumor tissue known as tumor stroma. The basic constitution of the tumor stroma includes the nonmalignant cells known as the stromal cells. Compared to nonsolid tumors, solid tumors pose distinct challenges to chemotherapy owing to the physical and biochemical complexity of their local environment, commonly referred to as the tumor microenvironment. Compared to normal tissue, tumor tissue has distinct structural properties that often hinder the delivery and distribution of anticancer drugs throughout the tumor mass and limit the efficiency and efficacy of drug treatment. Thus, understanding the detailed structural characteristics of a solid tumor with its microenvironment is indispensable for developing new treatment strategies.

### 2.1 Tumor Stroma

The tumor stroma is the abetting structure of tumor tissue. It is composed of non-malignant cells of tumor-like cancer-associated fibroblasts (CAFs) (Raghu et al., 2016; Tang et al., 2016; Lee et al., 2018; Liu et al., 2019; Monteran and Erez, 2019; Wang et al., 2019; Yavuz et al., 2019), tumor-associated macrophages (TAMs) (Qian et al., 2009; Noy and Pollard, 2014; Larionova et al., 2019; Lin et al., 2019), tumor-associated neutrophils (TANs), mesenchymal stem cells and extracellular matrix (ECM) with fibrous structural proteins (e.g., collagen and elastin), fibrous adhesive proteins (e.g., laminin and fibronectin), and proteoglycans. Nests of malignant tumor cells are linked through junctional proteins (e.g., claudins, desmoglein-2, and E-cadherin) in most solid tumors derived from epithelial tissues. These nests are surrounded by tumor stroma which plays a key role in regulating the behavior of cells found in the local milieu. Tumor stroma creates a niche that aids in seeding metastatic cells and intervenes in drug delivery to tumors. It generates a physical obstacle of stroma proteins that restrict drug penetration and connection between drug candidates, tumor-infiltrating immune cells, and target receptors present in the tumor cell surface. Tumor stroma generates cytokines and chemokines, which prompt synthesis of stroma proteins, prevent activation of immune cells, and activate immuno-suppressive cells such as regulatory T cells. Stroma is associated with characteristic tissue development and homeostasis in the tumor microenvironment, distinct from those associated with normal tissue (Kim et al., 2013; Nunes et al., 2019). Moreover, ECMs produced in most tumors make them more rigid and different types of collagen molecules are also accumulated, forming a thick network inside tumor tissue, resulting in a decrease in pores of tumor matrix, which restrict tumor penetration of nanomedicine useful for therapy. Amplified rigidity increases interstitial fluid pressure, which further restricts the distribution of nanomedicine throughout tumor mass. Therefore, targeting genetically stable stromal cells provides an additional benefit.

### 2.2 Tumor Stromal Cells

Stromal cells exhibit constant synthesis and release of growth factors, connective tissue components, and cytokines, cooperate with malignant cells to proliferate, invade, and metastasize, which seek major attention in tumor-targeted drug delivery using nanomedicine (Choi et al., 2013; Raghu et al., 2016; Tang et al., 2016; Lee et al., 2018; Liu et al., 2019; Monteran and Erez, 2019; Wang et al., 2019; Yavuz et al., 2019). Cancer-associated fibroblasts (CAF) are major cells found in tumor-associated stroma compared to stroma cells in healthy tissue (Kalluri, 2016). CAF are spindle-shaped mesenchymal cells characterized by constant activation, faster proliferation, and accumulation of large amounts of ECM compared to fibroblast in normal tissue (Kim and Bae, 2016). Tumor cells and stromal cells upregulate different types of profibrotic growth factors in the tumor microenvironment to transdifferentiate stromal fibroblast in CAF. They release various growth factors, for instance, epidermal growth factor (EGF), hepatocyte growth factor (HGF), and insulin-like growth factor-1 (IGF-1) and affect cell proliferation, invasion, and metastasis. They are involved in angiogenesis along with inflammatory cell recruitment. CAF are also engaged in the arousal of angiogenic processes and engages more inflammatory cells. Another very important cell type present in tumor stroma is tumor-associated macrophages (TAMs), which are immune cells of the tumor microenvironment. TAMs suppress antitumor immune responses, generate an immune suppressive microenvironment, and control tumor-associated changes in ECM by secretion of profibrotic growth factors. They produce cytokines (IL-I and IL-8), tumor necrosis factor-α (TNF-α), growth factors (EGF, HGF, bFGF, and VEGF), and various enzymes (Li et al., 2017a). TAMs regulate cancer stem cell activities in solid tumors. Tumor-associated neutrophils (TANs) are also a dominant form of immune cell infiltrates, found in various types of cancer (Masucci et al., 2019; Wu et al., 2019). They generate neutrophils, reactive oxygen species, cytokines, growth factors, and proteinases and play key roles in controlling tumor cell proliferation, metastasis, angiogenesis, and antitumor immune suppression (Masucci et al., 2019; Wu et al., 2019).

### 2.3 Characteristics of Solid Tumor that Influence Nanomedicine Penetration

**FIGURE 3:**
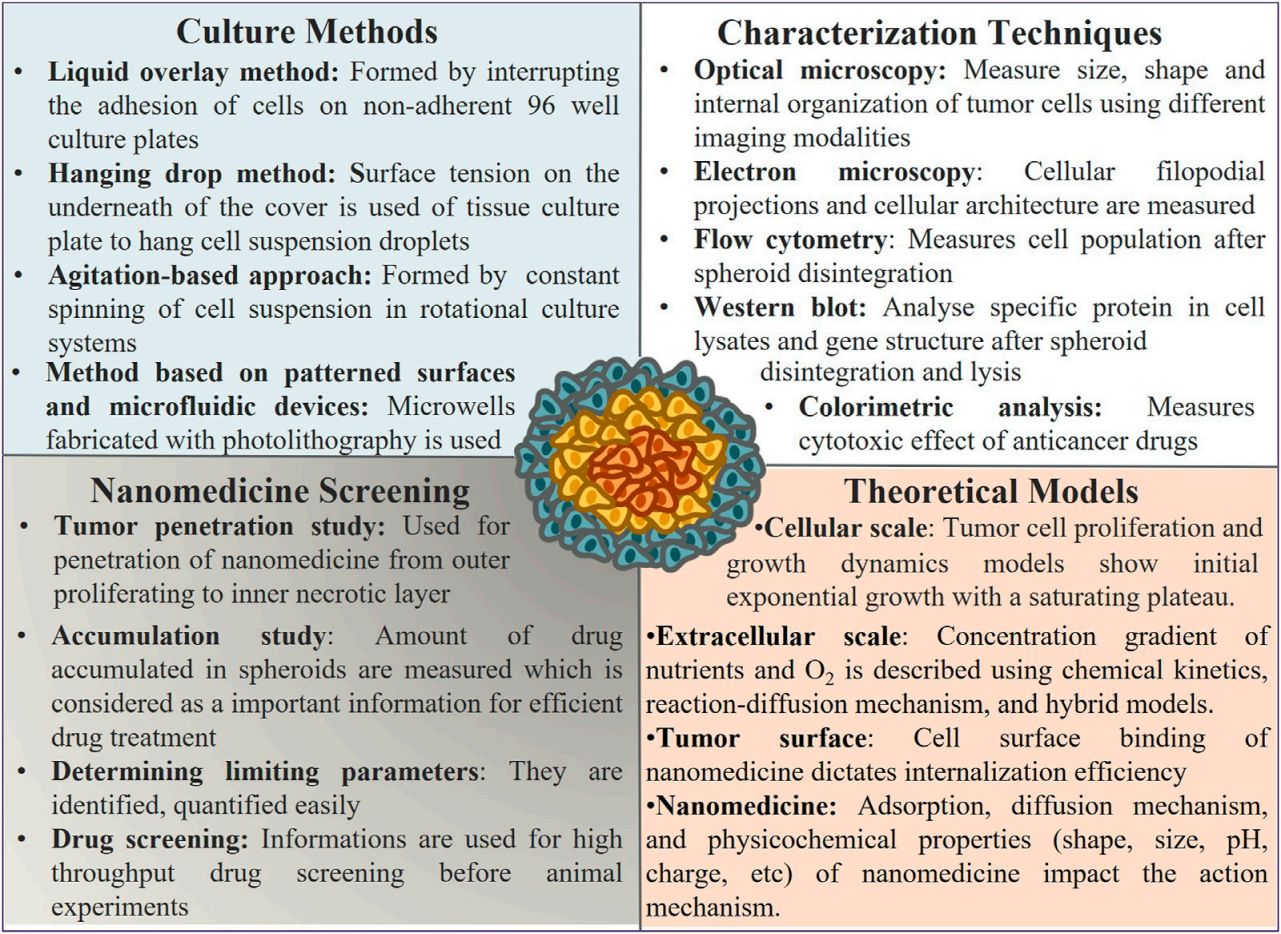
Tumor spheroid culture, characterization, use, and parameters that affect its activity as a model system: tumor spheroids are prepared using different experimental methods, and various techniques are used to characterize their architecture, properties, and application for nanomedicine screening. Several parameters affect the structure, growth, and efficiency of nanomedicine screening, as revealed from the theoretical study.

Nanomedicine facilitates the transport of drug candidates from tumor surface to center. The penetration across tumor mass gets occasionally influenced by the specific properties of tumor architecture (Figure 3).

#### 2.3.1 Abnormal Vasculature

The vasculature in solid tumors are heterogeneous, which leads to perfused areas, and results in abnormal blood flow in the tumor (Tong et al., 2004; Danquah et al., 2011; Mizukami et al., 2012). Tumors can be sustained via the angiogenesis process to generate new blood capillaries and permit oxygen and nutrient transport by diffusion (Mark et al., 2013; Li et al., 2018; Lugano et al., 2020). Healthy tissue forms new blood capillaries and regulates a sufficient amount of blood supply for cells, whereas the new blood capillaries in tumor tissue are poorly organized and heterogeneous in nature. Due to this abnormal vasculature, some parts of tumors are not easily accessible to demonstrate the therapeutic outcomes (Tong et al., 2004; Danquah et al., 2011; Mizukami et al., 2012). Normalization of tumor vasculature is one of the theories behind using anti-angiogenic drugs, which makes it more accessible for chemotherapy (Goel et al., 2011). However, in general, this abnormal and leaky tumor vasculature with respect to healthy tissue vasculature allows nanomedicine to be distributed in the tumor region and the delivery of drug candidates by the well-known enhanced permeability and retention effect (EPR effect) (Greish, 2007; Fang et al., 2011; Maeda, 2012; Maeda et al., 2013; Prabhakar et al., 2013; Maeda et al., 2016). Moreover, the impaired lymphatic drainage of tumors allows nanomedicine to be retained over there for a long time, again fostering the sustained release of drugs. EPR effect allows nanomedicines to not touch healthy tissue and thus exhibit low therapy-related undesired toxic side effects (Greish, 2007; Fang et al., 2011; Maeda, 2012; Maeda et al., 2013; Prabhakar et al., 2013; Maeda et al., 2016).

#### 2.3.2 Elevated Interstitial Fluid Pressure

Healthy tissue regulates interstitial fluid pressure in a way where the total pressure gradient between the tissues and the blood vessels enhances fluid flow and nutrient transport out of blood capillaries and into the cells. However, in tumor mass, there is abnormal vasculature with an increased interstitial fluid pressure along with high cell density and impaired lymphatic drainage (Heldin et al., 2004). This increased interstitial fluid pressure within the tumor causes inefficient uptake of nanomedicine. Various antagonists of vascular endothelial growth factor, antifibrotic agents, and transforming growth factor-beta are commonly used to decrease the interstitial fluid pressure to improve the transport and penetration of nanomedicine within the tumor mass.

#### 2.3.3 Dense Extracellular Matrix

The dense extracellular matrix is a significant barrier to the nanomedicine transport to reach tumor cells (Netti et al., 2000). Low blood supply is the consequence of abnormal vasculature within tumors. Drugs are transported by diffusion due to the insufficient convective transport within tumors. Fibrous macromolecules (collagen and glycosaminoglycans) fill in the extracellular spaces in solid tumors, resulting in a relatively dense extracellular space in solid tumors compared to the healthy tissues as the collagen content is significantly higher in solids tumors than in the normal tissues (Lu and Weaver 2012; Hynes, 2009; Frantz et al., 2010; Kim et al., 2011; Lu and Weaver_2012; Eble and Niland, 2019). There is no hindrance in the diffusion of small drug molecules through this protein matrix, but impaired mobility is observed in the case of large size of nanomedicine, resulting in them being confined in the areas surrounding the blood vessels (Greish, 2007; Fang et al., 2011; Lu et al., 2012; Eble and Niland, 2019). The impaired transport of nanomedicine through the dense extracellular matrix could be overcome by the degradation of extracellular matrix proteins.

### 2.4 Solid Tumor Models

**FIGURE 4:**
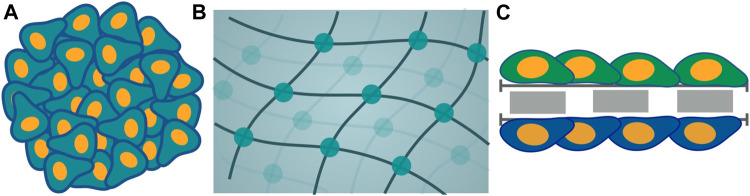
Schematic representation of different models of cell culture is categorized into two classes: non-scaffold-based and scaffold-based. Non-scaffold-based culture is multicellular tumor spheroid **(A)** and scaffold-based cultures are hydrogel **(B)** and inserts **(C)**.

Among the plethora of models for tumor cell culture consisting of the monolayer, 2D, and 3D culture, there is a foremost requirement of models congruent with intended efficient pathways of drug delivery (Waite and Roth, 2012). Different tumor cells are cultured as *in vitro* models to understand the underlying physical mechanism and chemical basis for the biological phenomena exhibited during tumor growth. The 3D tumor spheroids models with complex physiology and the microenvironment as a real solid tumor are highly persuasive. Various criteria take heed for the appropriate 3D cell culture model for targeted drug delivery with impaired efficacy. The 3D cell cultures are obtained mainly in two categories: non-scaffold-based cell cultures and scaffold-based cell cultures (Figure 4). For both non-scaffold and scaffold-based cell cultures, ECM and drug resistance are the two most primitive properties essential to be incorporated into an *in vitro* model of tumors. ECM is the non-cellular component of tissues, which acts as the link that establishes cell-cell communication for interaction among themselves and induces growth as a unit for spheroid culture. ECM components for scaffold-based cell cultures are natural like collagen, semi-synthetic like chitosan, or synthetic biomaterial like polycaprolactone (Costa et al., 2016). The obstruction to continuous flow and transport of drug molecules across the ECM, caused by the rise in interstitial fluid pressure, is known as drug resistance. Thus, this network plays an indispensable role in the proper channelizing of drug molecules to the core of a tumor with minimum resistance. Considering the impact of ECM, various kinds of scaffold and non-scaffold-based cell culture models have been developed. Scaffold-based 3D cell culture models are hydrogels and inserts, in which cells are grown embedded into platforms that mimic the ECM architecture. Hydrogel is a crosslinked polymer network (Ghosh et al., 2014; Ghosh et al., 2016). It is a colloidal gel with water as a dispersive medium. Hydrogel is cells seeded in gel-based 3D structures. For hydrogel to be used as an *in vitro* cell culture model, it should reflect a higher rate of reproducibility, which involves constructing the same results agreeing with the original study with the higher precision when produced again. In the field of biomedical research, where applications of *in vitro* cell cultures are widely increasing, reproducibility becomes one of the key components for establishing consistent results similar to *in vivo* models. Hydrogels lack reproducibility and ECM components impart resistance to drug penetration by diffusion (Achilli et al., 2012; Costa et al., 2016). Materials, such as collagen, that are used to mimic the ECM components are expensive. Hydrogel restricts the penetration of compounds leading to cell isolation for analysis, thereby losing its ability to capture spatial information (Sant and Johnston, 2017b). Thus, this restricts its use for a more useful cell culture model. On the other hand, inserts are another 3D cell culture scaffold-based model. This cell culture system consists of two parts: a plate as a scaffold with wells and insert. Inserts are like porous membranes anchored to the platform such that they will allow the nutrient media transport to them by attaching their surface to the cells regulating their growth and exchange through membranes for the transport study. More precisely, they are like cells seeded in structures constituted by different biomaterials like polycarbonate. These biomaterials may have similar properties as those of ECM. However, ECM components create the barrier that brings forth the resistance to drug flow, providing hindrances to the drug penetration. They have deficits in reproducibility, majorly dependent on the methods used for the scaffold fabrication. Thus, using these two models, hydrogel and inserts are useful but at the cost of extracellular matrix and drug resistance, which are the foremost priorities for the most efficient targeted drug delivery.

Cell culture models that are non-scaffold-based are the self-assembled aggregates of cells, such as multicellular tumor spheroid. ECM in these cell cultures is composed of protein produced by cells during the formation of cell culture (Costa et al., 2016; Han et al., 2021). Highly dense ECM components are the driving force for impaired drug penetration without any resistance. Both of the fundamental requirements are accompanied. Thus, the facets that play a pivotal role are extracellular matrix and drug resistance leading to one of the appropriate platforms for non-scaffold-based multicellular spheroid as cell culture model (Costa et al., 2016). The 3D spheroid models took precedence over all other existing models, including dimensionality of 2D and 3D models (Table 1).

**TABLE 1:** Comparison of different 3D culture cellular models.

Model	ECM	Drug penetration	Cellular heterogeneity	Cellular organization	Gene expression
Spheroids	The deposition of this connective network is similar to the *in vivo* tumor	A highly dense connective network is responsible for impaired drug penetration	Cancer cells cultured with fibroblast, immune cells, and endothelial cells lead to heterogeneity	Spheroid of three zones: proliferating, quiescent, and necrotic varying in proliferating rate and gradient of oxygen, nutrients, waste accumulation, and CO_2_	Showing similarity as the *in vivo* tumor
Hydrogel	Artificial and may have some components which are present in the native matrix	Barriers created by the connective network may be responsible for resistance to drug penetration by diffusion	Varying cell types can be grown on the scaffold	It is spontaneous and consists of heterogeneous cells. The necrotic layer may be formed	Resembles with *in vivo* tumor
Inserts	Consists of biomaterials having similar properties as ECM	Barriers established by ECM may lead to some resistance to drug penetration	Heterogeneous cells can be grown on the platform	The organization of cells is spontaneous and embedded with cellular heterogeneity. The innermost core consisting of necrotic cells may be formed	Resembles with *in vivo* tumor

The foremost properties of the cell culture models have been discussed, paving the path for the understanding of factors on which the drug delivery mechanism of spheroid depends. The delivery competence of therapeutic drug molecules is highly dependent on the tumor architecture. Modeling the internal structure of a tumor is a matter of greater importance for the impaired efficacy of drug delivery. Spatiotemporal study of the growth of tumors is the point of prime focus primarily influenced by the complex compartmentalized tumor architecture. Consequently, for the transport and penetration of the drug molecules to the core, an insight into the modeling mechanism can state the benefit of tailoring and governing treatments. Thus, to understand the fundamental mechanism for the detailed study of critical factors admissible for targeted drug delivery, mathematical modeling of a tumor is required.

The following study will comprise a broad view of both key components, tumor and nanomedicine. The former discusses the target itself, multicellular tumor spheroid as a tumor *in vitro* model, its architecture, factors assisting cell-cell interaction, its culture methods, the characterization of tumor spheroid, and the mathematical models supporting its growth at various cellular scales. Moreover, the latter discusses the drug molecules to be treated on the target, consisting of the study of key attributes of nanoparticles affecting their efficient delivery to target and the mathematical models supporting their adsorption, internalization, and diffusion at distinct scales.

## 3 Tumor Spheroids

Multicellular tumor spheroids are the 3D architecture of cancer cells that potentially reflect the *in vivo* conditions of tumors in the body. They can be cultured with only cancer cells or co-cultured with cancer cells and other cell types under various conditions (Nunes et al., 2019). Just as in naturally occurring tumors, these tumor spheroids also develop similar properties, which provide insightful details to study them an important model in cancer research, bridging the gap between *in vitro* cancer cell line cultures and *in vivo* tumors. The 3D architecture of miniature cellular aggregates modeled in a tumor spheroid is widely used for studying different types of cancers *in vitro*.

### 3.1 Structure of Tumor Spheroids

**FIGURE 5:**
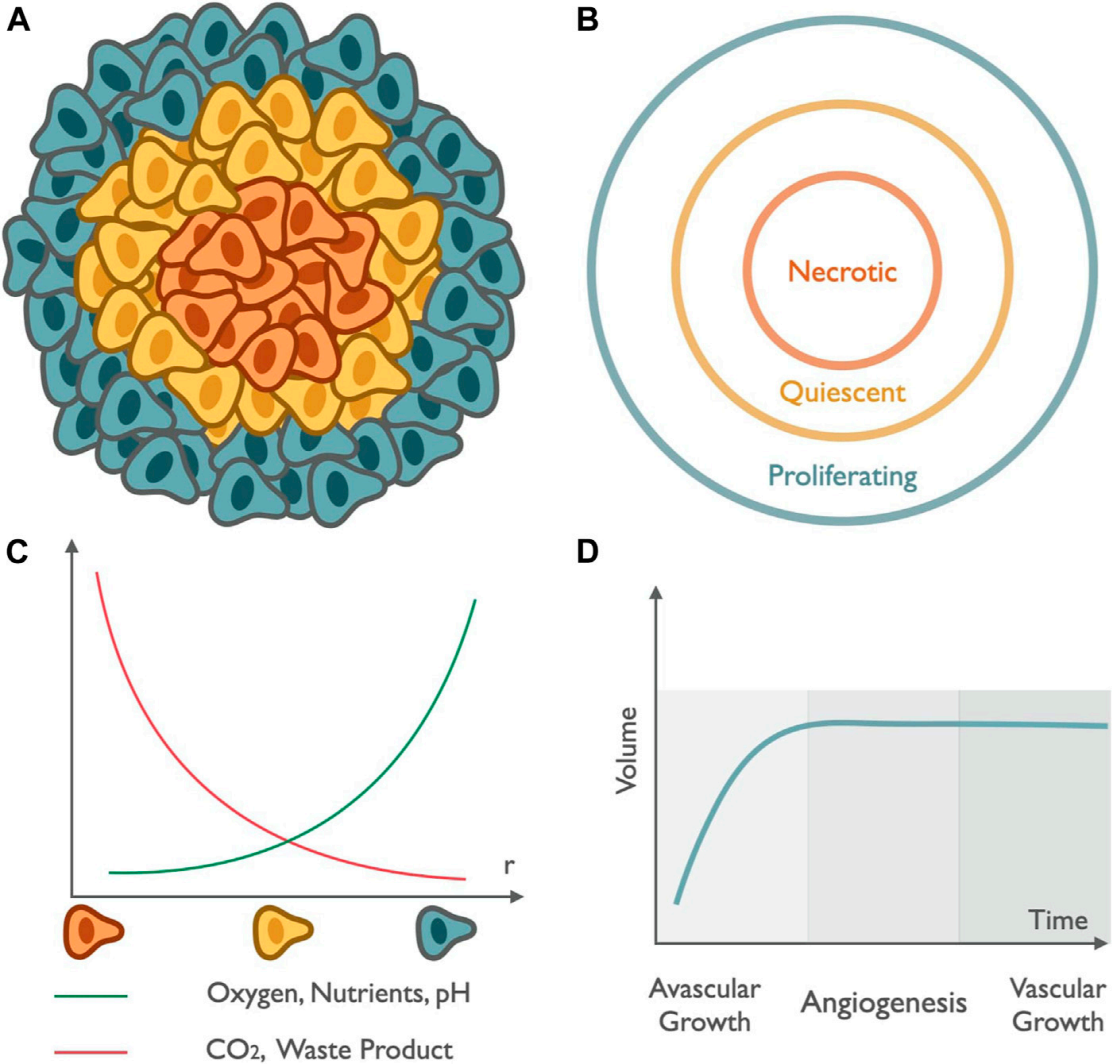
Tumor spheroid structure and growth: Schematic **(A,B)** represents the compartmentalized structure of tumor spheroids. The peripheral region in green represents the outermost zone, rich in oxygen content, and responsible for the rapid multiplication of cell numbers known as the proliferating zone. The annular region in yellow depicts the region with depletion in nutrients and oxygen known as the quiescent region. The innermost part in orange highlights the zone with the lowest pH and also deficits in oxygen, leading to the death of healthy cells known as the necrotic zone. **(C)** The graph in green represents the increase in the availability of oxygen, nutrients, and pH and the curve in red represents the variation of CO_2_ content and waste accumulation as we move radially outward from the center. **(D)** Growth curve: spheroid growth can be described in two different stages, initial avascular growth, where the volume of the tumor increases exponentially, followed by a dormant phase of saturation till the growth of spheroid reaches a plateau, and the transition from avascular growth to vascular growth occurs via angiogenesis, which represents the initiation of formation of new blood vessels led by a vascular growth dominated by the process of growth of cells rich in blood vessels that provide them with a gateway through which they enter metastasis to distant sites.

Multicellular tumor spheroids are spherical *in vitro* self-assembly of cellular aggregation representing the characteristics of *in vivo* solid tumors. In this self-assembled organization, cells aggregate, sort, and compartmentalize to separate different regions of the spheroid (Nath and Devi 2016). To impersonate the shear properties of the *in vivo* model of solid tumors, the study of the internal structure of the *in vitro* model of a spheroid is essential. The internal structure is incorporated with different cell layers based on the concentration gradient of nutrients, oxygen to regulate cell function, differential zones of proliferation and growth factors, pH, and cellular density. All these factors of the cellular organization play an indispensable role in studying the diffusion and penetration for impaired nanomedicine delivery (Lazzari et al., 2017; Sant and Johnston, 2017b; Costa et al., 2016). In accordance with differential proliferation rate, the structure can be broadly categorized in three different zones: proliferation, senescent, and necrotic zones; see Figure 5 (Mehta et al., 2012; Lazzari et al., 2017; Sant and Johnston, 2017). Modeling of spheroid growth can be done by taking into account the following spheroid cellular organization. These layers are characterized by the decreasing gradient of nutrients, oxygen, and pH, from the exterior to the center of the spheroid and by the increasing gradient of CO_2_, lactate, and waste from the exterior to the center of the spheroid. The outer layer where cells resurge and escalate rapidly in number is called the proliferation zone. It exhibits high proliferation rates in the spheroid periphery. The proliferation is stimulated by the constant exposure of the cells to oxygen and nutrients. When the oxygen diffusion and nutrient availability become a limiting factor, the cell’s proliferation rate decreases, giving rise to the middle layer made up of senescent cells. In the middle zone, cells can no longer divide due to the depletion of nutrients; nevertheless, they are active and alive, which is identified as the senescent zone. The middle region is followed by a decrease in cell metabolism as the distance from the outer region increases. The supply of nutrition is also depleted as we move from the periphery to inner zones. Finally, the core region, characterized by depletion in oxygen concentration, results in hypoxia, nutrient supply, and waste accumulation leading to a critical situation of cell necrosis (Figure 5). It is identified as the region where cells are noxious and lead to death, known as the necrotic zone. This core is the zone of the lowest pH (6.6–7.2) within the tumor spheroid. In this hypoxic environment, the pyruvate is converted into lactate by the cancer cells to obtain energy, known as the Warburg effect. The accumulation of lactate results in an acidified core of the spheroid and makes it favorable for drug release from nanomedicine.

The annular organization of subsequent cell layers within the spheroid is reported to be the key factor behind the inadequate therapeutic efficacy of anticancer drugs, as the presence of cells in the successive region from the outer zone, that is, the quiescent and necrotic region, reduces the potency to penetrate nanomedicine. In the hypoxic region of the spheroid, different drugs that cause cell death via the formation of reactive oxygen species (ROS) have a very low therapeutic efficacy (Liang and Liu, 2016; Al-Akra et al., 2019). Moreover, many drugs (e.g., paclitaxel) that promote cytotoxicity to the cells and proliferation have a poor impact in this innermost zone and that is why even the drugs which are highly effective in rapidly multiplying cells have a poor therapeutic benefit in the interior layers of the spheroid for the same reason. Moreover, low environmental pH catalyzes the change of net charge of drug molecules (e.g., melphalan, methotrexate, and mitoxantrone), tumor penetration, and intracellular uptake. The typical low pH condition at the interior zone induces the cancer cells to start expressing different kinds of hypoxia-inducible factor (HIF) family, which promotes the proliferation and consequent survival rate of the cancer cells (Masoud and Li, 2015; Albadari et al., 2019; Hompland et al., 2021).

### 3.2 Characteristics of Tumor Spheroids

The application of multicellular tumor spheroid in tumor-drug delivery is increasing rapidly. The key characteristics of a promising *in vitro* spheroid model are to accurately mimic the *in vivo* solid tumor, incorporating the real biological characteristics such as heterogeneity, microenvironment, and cellular arrangements. Spheroid is a well felicitous *in vitro* model for the aimed conveyance of nanomedicine due to its ability to transcribe the intrinsic characteristics found in solid tumors. We will elucidate the following characteristics of the spheroid in detail such as cellular heterogeneity, cell-cell signaling, internal structure, ECM deposition, gene expression, and how the external modification to spheroid could lead to efficient drug delivery, and how mathematical modeling approaches could give us the tools to decipher the underlying mechanisms (Albrecht et al., 2006; Ekert et al., 2014; Popilski and Stepensky, 2015; Shamsi et al., 2019).

Three-dimensional multicellular tumor spheroid is widely used as a model system for studying different types of cancers and screening of nanomedicine efficacy. Cellular heterogeneity is one of the critical aspects that is needed for this study and represents the diverse nature of cell types showing distinct morphological and phenotypic profiles. Cellular heterogeneity is embodied with the multiple cells that vary in their protein and gene expression. Spheroid is embraced with different heterogeneous tumor and stromal cells with varying ratios that lead to its cellular heterogeneity. The *in vitro* model can be established with homotypic cancer cells only or heterotypic cancer cells cultured with fibroblast, immune cells, or endothelial cells (Costa et al., 2016; Lazzari et al., 2017; Millard et al., 2017). In heterotypic tumor spheroids, the number of cells could be varied to closely mimic heterogeneity in solid tumors. Moreover, interacting networks among cells having this heterogeneity are demanded. Signaling established between the cells which are in close proximity helps in reproducing the spheroid’s key aspects and its microenvironment. All the cells growing in close proximity provide a route to a highly interactive microenvironment for physical communication and signaling pathways of cells to drugs. This heterogeneity and signaling can help decipher how multiple cell types might impact drug delivery to the site.

Solid tumors exhibit complex cellular microenvironment and architecture, where extracellular matrix (ECM) plays a crucial role (Lu et al., 2012; Pickup et al., 2014; Bussard et al., 2016; Lazzari et al., 2017). ECM is an essential component for binding the cells together via proteoglycans and fibrous proteins. The extracellular matrix (ECM) compositions, e.g., fibronectin, laminin, proteoglycans, collagen, and tenascin, are deposited by cells within 3D spheroids like solid tumors (Frantz et al., 2010; Kim et al., 2011; Lu et al., 2012; Hynes, 2013). The ECM (α5- and β1-integrin) in the tumor spheroid form a barrier known as limited mass transport, further limiting the insertion and uniform distribution of nanomedicine in the solid tumor mass. Extracellular matrix components in a highly interactive 3D microenvironment of spheroids make the structure more compact by depositing the protein, leading to an increase in the interstitial fluid pressure and further limiting impaired therapeutic efficacy of nanomedicine. It helps tumor cells in regulating different cell functions and maintaining the complex cell network. ECM-related signaling pathways play a crucial role in tumor progression, cancer cell migration, and penetration (Frantz et al., 2010; Kim et al., 2011; Lu et al., 2012; Hynes, 2013). Tumor cells within solid tumors grow in two stages. Initially, the tumor grows very rapidly, termed the avascular growth phase. Then, cells continue to stay in a dormant phase followed by vascular growth with the generation of new blood capillaries (angiogenesis) promoted by pro-angiogenic factors (e.g., angiogenin) and ECM rebuilding mediated by proteases (e.g., MMP-2 and MMP-9). Similarly, the progress of a tumor in an *in vitro* model is the collective dynamics of interaction between tumor cells and their microenvironment. The initial volume growth increases exponentially with time until it reaches a certain value (∼400 μm in diameter), but later the growth of volume decreases with time and becomes constant, referred to as the spheroid growth plateau (Figure 5). This avascular tumor expansion, growth plateau, and vascular expansion as spheroid growth varying with both temporal and spatial dimensions can be modeled mathematically.

Growth factors and the cellular ECM protein can be encoded with the learning of genes. Gene expression is a closely constrained process regulating the response of a cell to its changing environment. The study of gene expression is vital as it involves the conversion of DNA’s instruction into functional products like protein (Costa et al., 2016). Gene expression profile is firmly affected by the cellular organization of the spheroid. The abundant target protein expression, also known as overexpressed gene, plays an essential role in studying cancer progression, invasion, and metastasis. There are various factors that influence the morphology of tumor spheroids, such as cell type, cell density, culture media, method of culture, and mechanical stress. Based on their compactness, spheroids could be compact spheroid, tight aggregates, and loose aggregates of cells. In a compact spheroid, cells are tightly bound to each other. In tight or loose aggregation, cells do not form a complete sphere and can be easily disintegrated. Aggregation of cells initially occurs by loose bonds between integrin and ECM, then forming close contact through N-cadherin to E-cadherin interactions. As the cell communication pathways, morphology, and polarity of the cells in solid tumors closely resemble the structure of multicellular tumor spheroids in many ways, the study of using tumor spheroids is a very important *in vitro* model in recent times for studying the penetration profile of nanomedicine and calculation of accumulation of anticancer drugs (England et al., 2013). Thus, cellular arrangements and internal structure in tumor spheroid closely resemble solid tumors, making it an appropriate model for the study of tumor growth and invasion as metastasis with the effect of drug candidates for screening of different nanomedicine formulations towards its efficacy and efficiency of disease management.

### 3.3 Culture Methods of Tumor Spheroids

A plethora of techniques to culture *in vitro* models are available, incorporating the use of cell attachment resistant surface forces to induce cell-cell interactions and ultimately support the formation of multicellular tumor spheroid (Figure 3 and Table 2). These techniques will be discussed in the subsequent part (Ishiguro et al., 2017; Lazzari et al., 2017).

**TABLE 2:** Tumor spheroid used as *in vitro* model for nanomedicine penetration study.

Sl no	Nanomedicine	Spheroid properties	Penetration details	Techniques	Comments	References
1	Doxorubicin-loaded NM coated with CD47 peptides (DOX/sNDF-CD47) of sub-150 nm size	Coculture of tumor-associated fibroblast MRC-5 cells with MCF-7 cells	sNDF-CD47 penetrate deeper into TS compared to control NM	CLSM	CD47 peptide assist penetration	Mo et al. (2019)
2	PG-co-PCL dendritic NM loaded with gemcitabine of 166 nm size	TS of MIA PaCa-2 pancreatic cancer cells. 150,000 cells per TS were used	NP carried gemcitabine 40 µm deeper into TS	CLSM	No ligand has been used for spheroid penetration	Ray et al. (2019)
3	pH-responsive crosslinked nanogels of 200 nm size loaded with cisplatin	TS of A549 cells with 200–300 μm size having 5 × 10^5^ cells	Nanogel located at 50 μm depth	CLSM	No ligand has been used for spheroid penetration	Cheng et al. (2019)
4	PLGA NP encapsulated with tetrandrine and a magnetic material (Fe_3_O_4_) with a size of 199 nm and a negative surface charge	TS with A549 cancer cell prepared by liquid overlay method with 250 μm size	NP penetrate to 160 μm depth	CLSM	PLGA NP deeply penetrates A549 TS, exerts an antiproliferation effect, and induces apoptosis	Wang et al. (2019)
5	Transferrin targeted core-shell NM encapsulating sorafenib and doxorubicin, size of 110 nm	3D HCC spheroid with a size of ∼200 µm	Penetration of targeted core-shell NP throughout the tissue causing uniform cell killing	CLSM	Transferrin assists in spheroid penetration	Malarvizhi et al., 2014
6	HPMA copolymer-based NM carrying pirarubicin of size 25 nm	Colon carcinoma C26 tumor cells (250–300 µm) and glioblastoma U87-MG cells (450–550 µm) were employed	C26 and U87-MG spheroids were observed with 120 and 80 µm penetration, respectively	CLSM	HPMA NM assists penetration of THP	Kudláčová et al. (2020)
7	Paclitaxel-loaded polymeric micelles with size 90 nm	The NCI/ADR-RES multicellular spheroids with 400–600 μm size were established by the liquid overlay method	The penetration capability of micelles was greater than control groups	CLSM	MMP2-sensitive peptide linker assists in tumor penetration	Yao et al. (2017a)
8	Pluronic NP and PEO-PPO-PEO triblock copolymers micelle	HeLa and U87 cells were to prepare TS of 500–600 µm size	Penetration NP observed at 80–100 µm depth	CLSM	Penetration depends on the transcellular transport of the carriers	Arranja et al. (2016)
9	Doxorubicin immobilized AuNC-cRGD-Apt NP	U87MG cells were used by a liquid overlay method of the diameter of 500–600 μm	NP located at 80 µm inside TS	CLSM	Targeting ligand cRGD assists penetration	Chen et al. (2016)
10	Theranostics thermosensitive micelle CuS functionalized by (PAAmAN−PEG) with size 8.9 nm	MDA-MB-468 cells with the size of 500 μm were formed using the hanging drop method.	NM shows a higher and homogeneous distribution in the central area of the tumor spheroids	CLSM	Targeting ligand facilitates the penetration of NP into tumor spheroids	Chen et al. (2017)
11	Gold NP coated with tiopronin with a size of 2–15 nm	MCF-7 cells were used in the liquid overlay method with 600 cells per well	NP penetration occurred in a size-dependent manner, with 2 and 6 nm AuNPs able to penetrate deeply into tumor spheroid	Bright-field and dark-field microscopy	Colloidal gold NP shows great potential to overcome delivery limitations	Huang et al. (2012)
12	Paclitaxel-loaded Ft-NP with a size of 150 nm and delivered via neuropilin-1- and tenascin C-mediated specific penetration	U87 glioma TS having 5 × 10^5^ cells were used by liquid overlay technique	Ft-NP-PTX penetrated deeper into TS compared to control NM	CLSM	Ft peptide- (fused FHK and tLyp-1 peptide together via cysteine linkage) functionalization facilitated its deep penetration	Kang et al. (2016)
13	Micelle with paclitaxel and 40 nm size	4T1 cells were used in TS with 100 µm size	Deeper penetration and improved cellular internalization of NP was observed in tumor tissues at pH 6.8	CLSM	No ligand has been used for spheroid penetration	Ke et al. (2018)
14	Ce6 conjugated mPEG-PLA NP	Avascular A549 spheroid model of 400–500 μm size was prepared by liquid overlay method	NP located at 70 μm depth	CLSM	The small size and the negative surface of NP help in easy penetration into the spheroids	Kumari et al. (2020)
15	Hyaluronic acid grafted micelles encapsulating optimal molar ratio (1:1) of Gem-C12 and HNK, with 53 nm size	TS of U87MG cells with 200 µm size	NP located to a depth of 50 µm	CLSM	The enhanced penetration results from active endocytosis by CD44 on the U87 cell surface	Liu et al. (2018)
16	NP with a mesoporous silica nanoparticle (MSN)-supported PEGylated liposome yolk and CCM coating, with 180 nm size	MCF-7 MCSs were cultured and prepared using a lipid overlay system with 10^4^ MCF-7 cells	Penetration throughout TS up to a 23.3-fold increase compared to the penetration of membrane vesicles	CLSM	NP exhibited moderate rigidity, which was attributed to its yolk-shell structure, leading to an improved tumor penetration	Nie et al. (2020)
17	Lipid-core micelles and liposomes as nanocarriers for encapsulation and delivery of NCL-240, with 200 nm size	NCI/ADR-RES spheroids with a diameter of ∼550 μm	Micelles located up to a depth of 100 μm	CLSM	Transferrin targeting enhanced penetration	Pattni et al. (2016)
18	Nanoformulations of CUR and DOX with scFv-targeted micelles	Multicellular 3D cancer cell spheroids of U87MG were prepared by the liquid overlay method with 10^4^ cells	Penetration observed up to a depth of 70 μm	CLSM	Using GLUT-1 scFv as the targeting ligand resulted in higher cellular internalization and better penetration	Sarisozen et al. (2016)
19	Targeted Mesoporous iron oxide nanoparticles, encapsulated perfluorohexane, and paclitaxel, with a diameter of 160 nm	Three-dimensional TS models with 200 µm in diameter prepared by using a liquid overlay method	Drug concentration was observed in the deep regions of tumor cells	CLSM	MF-induced PFH gasification increased the NP penetration and accumulation in the TS	Su et al. (2015)
20	Raloxifene encapsulated with styrene co-maleic acid (SMA) micelle, with a diameter of 65.34 ± 30.89 nm	PC3 cells TS with 8,000 cells and of 400 μm in diameter	Micelle effectively inhibits cell cycle progression, increases apoptosis, and alters the integrity of TS models	CLSM	No ligand has been used for spheroid penetration	Taurin et al. (2014)
21	Paclitaxel loaded to MHI-HGC nano-micelle forming MHI-HGC-PTX with 230 nm size	4T1-3D spheroid of 200–300 μm in radius	MHI-148 Cyanine Dye Conjugated Nanomicelle showed high penetration ability in the tumor spheroid	CLSM	Heptamethine dye as a targeting ligand, optical imaging agent, and NIR photothermal stimuli assists on-demand drug release	Thomas et al. (2018)
22	Docetaxel-loaded hybrid micelles with DSPE-PEG and TPGS (TPGS/DTX-M), with a diameter of 17–24 nm	Tumor spheroids were formed with KBv cells using the hanging drop method, size of 400 μm	TPGS has served as a permeation enhancer and assisted in drug penetration in TS	CLSM	Folate-modified TPGS hybrid micelles promote effective delivery of DTX	Wang et al. (2015)
23	iRGD-modified nanoparticles loaded with ICG and TPZ, with a diameter of 112 nm	4T1 cells- multicellular TS with 400 μm diameter	Nanoparticles located at a depth of 89 μm	CLSM	Conjugated iRGD onto the surface of the nanoparticles improves their penetration in TS	Wang et al. (2018)
24	curcumin-loaded					
	VES-g-PLL micelles, exhibiting an ultra-small particle size of ca. 30 nm and positive Zeta potential of 19.6 mV	C6 spheroids, with a volume of 250 mm³, were developed using the liquid overlay method	Curcumin-loaded micelles located in deeper regions of TS	CLSM	Ultra-small size and positively charged surface, Cur-Micelles promoted deeper penetration. No ligand was used	Xu et al. (2017)
25	DOX-loaded CQDs-TPGS-TPP nano micelles, size <160 nm	MCF-7/ADR-derived spheroids with a diameter of 300–400 µm	NP penetrated to a depth of 120 µm	CLSM	DOX penetration efficiency improved via CQDs-TPGS-TPP/DOX nanomicelles	Zhang et al. (2017)
26	Silver NPs functionalized with polyethylene glycol and aptamer As1411, with a diameter of 18 nm	C6 glioma spheroid model	The penetration ability of the AgNPs functionalized with PEG and As1411 was superior to that of the AgNPs modified only with PEG	CLSM	As1411 effectively increased the tumor penetration of the NPs	Zhao et al. (2019)
27	Transferrin modified (PEG-PE)-based polymeric micelles containing paclitaxel and tariquidar, with hydrodynamic diameter ca. 16 nm	3D spheroids of SKOV-3TR cells, with a diameter larger than 600μm, hypoxic micro-regions, and a necrotic spheroid core	Tf-targeted micelles penetrated deeper layers of the spheroid	CLSM	The small size of the micelles and Tf-targeting improved TS penetration	Zou et al. (2017)
28	GANT61 and curcumin-loaded PLGA nanoparticles, with a size of 347.4 nm	MCF-7 3D spheroid with 3 ×10^4^ cells	NP are observed in the deep regions of TS and kills all the bulk tumor cells and CSCs together by targeting EGFR and Hh pathway	CLSM	No ligand has been used for spheroid penetration	Borah et al. (2020)
29	Hyaluronic acid-coated single-walled carbon nanotubes loaded with doxorubicin	MDA-MB-231 cell spheroids	NP penetrates deep to the center of TS and induces cell apoptosis	CLSM	HA can specifically recognize CD44 acts as a targeting ligand in nanoparticles and assists tumor penetration	Liu et al. (2019)
30	siBcl-2/Dox-TPGS-LPs, size of 210 nm	3D H22 tumor spheroids with 4×10^5^ cells	siBcl-2/Dox-TPGS-LPs exhibited better penetration compared to the control NM	CLSM	TPGS-modified cationic LPs assists in the penetration	Tan et al. (2019)
31	ND-PG-RGD composite loaded with doxorubicin to give the final product Nano-DOX, with a hydrodynamic diameter of 83.9 ± 32.3 nm	3D GC spheroids	Nano-DOX penetrated deeper layers of the spheroid	CLSM	TAM serves as a carrier and reservoir to release drugs to the TS	Li et al. (2017)
32	Polymeric hybrid nano micelles to co-deliver the Dox and microRNA-34a (miR-34a)	MDA-MB-231 3D multicellular spheroids (approximately 600–800 μm)	Suitable micelle size caused deeper penetration of Dox into the TS, generating efficient cell killing	CLSM	No ligand has been used for spheroid penetration	Xie et al. (2019)
33	MMP2-sensitive FR-targeted, DSB loaded polymeric nanoparticulate micelle with a size of 100–200 nm	NCI/ADR-RES multicellular spheroids a diameter of 400–600 μm	The polymeric micelle showed deeper penetration than the control NP	CLSM	Multifunctional micellar nanoparticles combined (MMP2)-sensitive tumor (site) targeting with folate receptor-mediated tumor (cell) targeting	Yao et al. (2017b)
34	Glycogen NPs for the therapeutic delivery of nucleic acids with a diameter of 20–150 nm	293T-Luc cells and PC3 cells were used for TS preparation	Glycogen constructs penetrate the spheroid ECM and are effectively internalized into the tumor cells	CLSM	The controlled size and surface charge density of glycogen-siRNA constructs minimized the interactions with serum proteins and allowed significant penetration	Wojnilowicz et al. (2018)
35	Nano-doxorubicin-loaded monocytes	U87 cell spheroids	Drug release from nano-DOX-MC was observed at deeper layers of the TS	CLSM	Nano-DOX can be effectively delivered by MC	Wang et al. (2018)
36	Small unilamellar vesicles (SUVs) probed with different lipid compositions, with a hydrodynamic diameter of approximately 85 nm	BxPC-3 and HPSC multicellular spheroids were prepared by lipid overlay method with 5,000 BxPC-3 and 5000 HPSC cells	Lip3 displayed the best penetration compared to the rest of the liposomes diffused into the MCSs	CLSM	Liposome mechanics is a design parameter for enhancing drug delivery in TS	Dai et al. (2019)

#### 3.3.1 Liquid Overlay Method

Spheroids are formed by interrupting the adhesion of cells on non-adherent 96-well culture plates, coated with poly-2-hydroxyethyl methacrylate or agarose, which prevents attachment (Costa et al., 2014). This method allows the culture of both homotypic and heterotypic spheroids where size and morphology could be changed easily by changing the number of cells used for seeding in individual wells. Additionally, ease of handling and production of a large number of spheroids makes this approach very useful for different types of high-throughput assessments. The method demands a lower volume of culture media and testing materials. However, plate to coat with poly-2-hydroxyethyl methacrylate or agarose takes a longer time. Commercially available pre-coated low adhesion plates increase the overall cost of the experiment.

#### 3.3.2 Hanging Drop Method

This method utilizes surface tension on the underneath of the cover of the tissue culture plate to hang cell suspension droplets (∼20–50 μL). Further gravity helps cell accumulation at the liquid-air interface (cover of drop), resulting in aggregation into a single spheroid. Both homotypic and heterotypic spheroid size could be controlled by changing cell density. This method is highly reproducible. However, the limited volume of seeding suspension does not supply enough nutrients for long-term culture. It required transferring to another culture plate for experiments, which affects the integrity of cells in the spheroids. It is an extremely time-consuming and labor-intensive process, which is not suitable for large-scale applications. Some commercially available plates are to be used for better outcomes (Lin and Chang, 2008; Benien et al., 2014).

#### 3.3.3 Agitation-Based Approach

In this technique, spheroid formation is done by a constant spinning of cell suspension in rotational culture systems that restore motion and support cell-to-cell interactions and avoid their attachment to the wall of the culture plate. The method provides control over the condition for large-scale production and long-term culture of tumor spheroids. However, controlling the number of cells per spheroid and their size is very difficult. Moreover, manual selection and transfer into different plates are necessary before further assay. Hence, it is labor-intensive and involves requiring a large amount of culture media, which limits their wide-scale application (Gianpiero et al., 2017).

#### 3.3.4 Patterned Surfaces and Microfluidic Devices

This technique utilizes arrays of microwells fabricated with photolithography. Low attachment surfaces are achieved by a coating of agarose or the use of non-adherent materials like polydimethylsiloxane. This method requires a limited number of cells, media, and reagents, making it suitable for high-throughput drug screening. Complexity is achieved with microfluidic devices displaying heterogeneous cell types. Various shaped channels ensure cell signaling and initiate the *in vivo*-like organization. The equipment required for this technique is expensive, hindering the wide application in the preclinical assessments of nanomedicines (Gianpiero et al., 2017).

### 3.4 Characterization of Tumor Spheroids

Advanced characterization techniques are utilized to characterize tumor spheroids based on, e.g., size, shape, cellular arrangements, protein and gene expression, cell cycle patterns, invasive nature, and metastatic potential of cancer cells to assess the nanomedicine (Figure 3 and Table 2). Various types of techniques are described as follows (Elizabete et al., 2016).

#### 3.4.1 Optical Microscopy

Bright field, dark field, differential interference contrast (DIC), phase contrast, and fluorescence microscope-based different imaging modalities are very important techniques for characterizing the size, shape, and internal organization of tumor cells in the spheroids (Table 2). An optical microscope is a more routine tool used to study the growth evolution and internal arrangements in each layer of tumor spheroids. Fluorescent microscopic techniques are commonly used to understand the amount of live and dead cells within spheroids, where calcein-AM and propidium iodide are routine stains used for the purpose. For histological analysis, hematoxylin and eosin assay, toluidine blue, and Masson’s trichrome are used. Fluorescence microscopy is a very important tool to check the therapeutic efficacy of nanomedicine equipped with various anticancer drugs with fluorescence properties (e.g., doxorubicin, epirubicin, and curcumin). This technique allows determining the drug penetration and distribution profile and calculating the amount of drug accumulated in the spheroids (Mikhail et al., 2014). Currently, more advanced techniques of confocal laser scanning microscopy (CLSM) are used to measure each layer’s penetration information (Zinchuk and Zinchuk, 2011; Rane and Armani, 2016). However, thick specimens are difficult to measure by CLSM where penetration of light is limited with water immersion objectives. Tumor spheroids are sliced into 5–10 µm thickness and used for measurements. To prevent distortion of tumor spheroid during sectioning, cryosectioning is commonly used with cryoprotecting agents. Penetration of the staining agent is not significantly hindered in sectioned slices but also in intact spheroids. Different fluorescence-based techniques such as light-sheet-based fluorescence microscopy (LSFM), two-photon microscopy, and multiphoton microscopy have been developed for imaging cell layers present in the interior of spheroids to avoid sectioning.

#### 3.4.2 Electron Microscopy

Electron microscopy-based technique is used to acquire images of spheroids with high magnification and resolution with cellular filopodial projections and cellular architecture involved in cell-cell physical interaction. Cell death after nanomedicine treatment is also studied using this technique. Scanning electron microscopy (SEM) with a high vacuum technique is most commonly used to prepare a sample following four stages of fixation. The initial spheroid is preserved and stabilized in order to allow its manipulation and imaging. In the next stage (dehydration), water in the sample is replaced with acetone or alcohol and processed for critical point drying where the sample is completely dried if any ethanol or acetone present in the sample is replaced by CO_2_, evaporated from the sample, and coated with sputter sample coating with gold for imaging. However, in the last two stages, disruption of the spheroid structure sometimes happens. To overcome these limitations, other advanced SEM techniques, such as low vacuum SEM and cryogenic SEM, are used as a substitution. Transmission electron microscopy (TEM) is another widely applied technique to evaluate nanomedicine penetration and accumulation in tumor spheroid. In this method, the spheroid is fixed chemically, dehydrated, and sectioned into thin slices, and before measurement, sections are stained with 2% uranyl acetate to generate more contrast.

#### 3.4.3 Flow Cytometry

Flow cytometry is used to determine the cell population in tumor spheroids, where individual cell analysis in suspension is performed after spheroid disintegration. Flow cytometry has widely been used to quantify the cellular uptake of nanomedicine and to evaluate their toxicity; but, however, it is a less efficient technique to understand nanomedicine penetration at different layers of spheroids as this technique requires the disaggregation of spheroids (Sasaki et al., 2020; Mo et al., 2013). However, flow cytometry is used for cell cycle pattern analysis in tumor spheroid where fluorescent dyes intercalate with DNA during the different stages of the cell cycle (especially for the proliferative and senescent zone) (Tindall and Please, 2007). Specifically, fluorescent dye interacts with DNA during S-phase that distinguishes senescent from proliferating cells. This type of fluorescent dye is used to identify cells in different phases of the cell cycle, like the S-phase and S-M phases. Fluorescent probes that target cellular components or proteins of interest also could be used for flow cytometry-based analysis of cell death and gene expression.

#### 3.4.4 Western Blot

Western blot is a very important technique that is widely used to analyze specific proteins in cell lysates and gene structure in tumor spheroids. Cellular homogenates are prepared from cultured spheroids after cell lysis in the presence of detergent. Particularly, the cell lysis process damage the cell structure and releases intracellular proteins from different subcellular compartments. Western blot allows detecting a low concentration of protein. However, the western blot is a semiquantitative method and that is why RTPCR is sometimes complemented with it. In this technique, gene expression is quantified through the synthesis of complementary DNA transcripts from RNA. This technique is used to identify different essential proteins in tumor progression and analyze therapeutic efficacy by assessing the expression of pro-apoptotic markers. This technique is used widely to check the efficacy of gene therapy in tumor spheroids.

#### 3.4.5 Colorimetric Analysis

The colorimetric analysis is based on the chemical assays used to measure cytotoxic effects of anticancer drugs. The colorimetric method is applied for assays like Alamar Blue acid phosphate, lactate dehydrogenase, MTS, MTT, and WST-8. These assays are based on the conversion of enzymes present in the subcellular compartment of live cells. Then, the formed product is determined by measuring the absorbance or fluorescence at specific wavelengths. Although colorimetric analysis is more applicable to monolayer culture, the spheroid system could be used after modification of experimental techniques. As a substitute for colorimetric assay, different spectroscopic techniques like tissue dynamic spectroscopy, Fourier transformed infrared imaging, and photon-induced X-ray emission (PIXE) are used less commonly to determine the toxic effect of drugs in tumor spheroids.

The next part of this review will address an overview of different types of mathematical models that have been developed to represent spheroid structure and how the insight drawn from these models helps in making an efficient drug delivery mechanism.

### 3.5 Mathematical Modeling of Spheroid Growth

The study of spheroid growth using mathematical modeling is more than half a century old (Laird, 1964). These quantitative approaches are invaluable tools for comprehending the cellular and transport phenomenon within spheroids and foreseeing the physiological acknowledgment to drug delivery. They can provide the pertinent perspective for drug delivery mechanisms and effectiveness in spheroid. Mathematical modeling of spheroid can be outlined into two distinct scales. First, the cellular scale describes cellular dynamics that lead to the model of tumor cell proliferation. The exact quantitative expression for this model is given by the Gompertz equation (Laird, 1964). It was commonly believed that tumors grow exponentially and stop until the host nutrition supply is exhausted. However, it has been observed that exponential growth is only dominant for a brief period of time and reaches a growth plateau as the tumor grows larger in size (Laird, 1964; Ward and King, 1999; Mehta et al., 2012; Costa et al., 2016). Secondly, on the other hand, the subcellular and extracellular scales describe the chemical dynamics with the help of the reaction-diffusion mechanism and hybrid models. Modeling tumor morphology starting from individual cells is usually divided into two categories incorporating both continuum and the cell-level description. Hybrid models deal with a combination of these two different descriptions. One is related to the periphery of the tumor, embraced with a cell-level description where it is advantageous to do so, and the other covers the two inner zones of tumor and the extracellular matrix pertaining to continuum description, i.e., cell population-based continuum models and individual cell-based discrete models. During the development of these models, the primary focus was on incorporating the different characteristics of spheroid growth, such as initial exponential growth and the concentration gradient of nutrients, oxygen, which are vitally important for the layered organization of spheroid and targeted nanomedicine delivery.

First, cellular dynamics can be studied mathematically by one of the pioneering models for tumor growth developed by Laird in 1964 (Laird, 1964). The growth kinetics of solid tumors is akin to spheroid, which can be classified into two levels. During the initial phase, the exponential growth of the tumor volume is observed. It is followed by a dormant phase of minimized metabolic activities until the spheroid growth plateau, where the spheroid’s volume attains a constant value (Figure 5). In improving the exponential model, the cell population growth curve with a time-dependent growth rate is considered. Let the size of the population at time t be W(t) and the growth rate decay exponentially be $a\left(t\right)=\alpha e^{-bt}$ . Here, the independent variable is time t , and the dependent variable is the tumor volume or population size W(t) . The corresponding ordinary differential equation for W(t) can be written as follows:

$$\frac{dW\left(t\right)}{dt}=\alpha e^{-bt}W\left(t\right).$$ (1)

The solution to this model shows tumor cell proliferation that can be expressed by a modified exponential process, commonly known as the Gompertz equation for sigmoidal growth, of the following form:

$$\frac{W\left(t\right)}{W_{0}}=e^{\frac{a}{b}\left(1-e^{-bt}\right)},$$ (2)

where W(t) is the tumor size at any time t , $W_{0}$ is the initial tumor size, and  b is constant. Now, $e^{-bt}$ can be expressed in power series as $\;e^{-bt}=\overset{\infty }{\underset{n=0}{\sum }}\frac{{\left(bt\right)}^{n}}{n!}$ . During the initial growth, i.e., at small t , $e^{-bt}$ can be approximated as $e^{-bt}\approx 1-bt$ , and the growth equation takes the simple exponential form $\frac{W\left(t\right)}{W_{0}}=e^{at}$ , which is consistent with the observation of initial tumor growth. At later times, the growth deviates from the pure exponential dependency and takes the Gompertz form. From the Gompertzian analysis, the theoretical upper limit of tumor growth for mice is typically $\approx 10^{9}$ cells, which is also consistent with the approximate size at death (Laird, 1964; Norris et al., 2006; Altrock et al., 2015; Costa et al., 2016).

In the above formalism of growth dynamics, there is only one independent variable time t , and the dependent variable is the volume of the tumor. Nevertheless, mathematical models have been developed using partial differential equations to study the spheroid growth and architecture at higher dimensions. For instance, a model has been developed to study quantities such as oxygen distribution which has more than one dependence, one is spatial and the other is temporal. One valuable characteristic of a spheroid is their limit of diffusion of about 150–200 μm for many molecules, specifically oxygen (Mehta et al., 2012). This diffusion limit gives rise to limited mass transport, as a result of which spheroid displays the gradient of oxygen, distribution of nutrients, metabolic waste accumulation, and proliferation profile inside them. Hence, a diffusion model for the oxygen concentration is requisite. The oxygen concentration in a tissue can be represented by ρ(r,t) , where the position r , in general, measures from the center of the spheroid at a time t. The reaction-diffusion equation for oxygen concentration inside a spheroid in the form of a partial differential equation can be written as follows:

$$\frac{\partial \rho \left(r,\;t\right)}{\partial t}=D\frac{\partial^{2}\rho \left(r,\;t\right)}{\partial r^{2}}-a\rho \left(r,t\right)-bN\left(r,t\right)\rho \left(r,t\right)+c\rho \left(r,t\right)\;,$$ (3)

where oxygen diffuses inside the spheroid with a diffusion constant D , decays with the rate constant a , and is produced with a rate constant c . The consumption of oxygen is proportional to the group size of the tumor cells N(r,t) and available oxygen concentration ρ(r,t) itself with a rate constant b .

A mathematical model is required to weave the insights gained from the discrete cellular dynamics into a coherent description of the reaction-diffusion mechanism. Combining the continuum model of growth-consumption as a reaction-diffusion model along with discrete cellular dynamics of cell growth and motility, a hybrid model has been developed. The model retains the cellular description in the rapid proliferation region on the periphery of the tumor and for the dynamics of tumor cell density, extracellular matrix (ECM) cells, matrix-degrading enzymes (MDE), and oxygen concentration as continua. The corresponding coupled dynamic equations of these individual quantities can be written as partial differential equations. For instance, a partial differential equation for the dynamics of tumor cell number $N_{T}$ is given by

$$\frac{\partial N_{T}}{\partial t}=D_{T}\frac{\partial^{2}N_{T}}{\partial r^{2}}-\frac{\partial }{\partial r}\left(N_{T}\frac{\partial N_{E}}{\partial r}\right),$$ (4)

where the diffusion constant of tumor cells is denoted by $D_{T}\;$ and extracellular matrix is represented by $N_{E}$ . The dynamics of ECM $\left(N_{E}\right),$ MDE $\left(N_{M}\right),$ and oxygen concentration (ρ) are represented by

$$\frac{\partial N_{E}}{\partial t}=-\phi \;N_{M}N_{E},$$

$$\frac{\partial N_{M}}{\partial t}=D_{M}\frac{\partial^{2}N_{M}}{\partial r^{2}}+\lambda \;N_{T}-\mu \;N_{M},$$

$$\frac{\partial \rho }{\partial t}=D_{\rho }\frac{\partial^{2}\rho }{\partial r^{2}}+f\;N_{E}-n\;N_{T}-c\rho \;.$$ (5)

The diffusion constant associated with ECM and MDE is, respectively, represented by $D_{E}$ and $D_{M}$ . The degradation of the extracellular matrix is directly proportional to the density of matrix-degrading enzymes and extracellular matrix with a proportionality constant ϕ . Matrix-degrading enzymes are produced by the tumor cells themselves with a rate constant λ and natural decay with a rate constant μ . On the other hand, the oxygen concentration is directly proportional to the ECM density with a rate constant f . It is consumed by the tumor cell at a rate n and decays at a rate c .

The hybrid model bridges two different types of models distinguishing between individual cell and cell population-based models. This model complements a fully continuous and complex description of tumor dynamics. The discrete cell interaction can be explained by a stochastic model, which is an off-lattice model. On a 2D lattice, probabilistic rules are applied to each cell pertaining to discreteness by defining stochastic reaction rates of respective events and may depend on the microenvironment (Kim and Stolarska, 2007; Altrock et al., 2015). In a hybrid model, these stochastic rates depend on the concentration of continuous variables ρ , $N_{M}$ , $N_{E}$ , and $N_{T}\;.\;$ Besides this, the hybrid model also specifies the guidance for processes like proliferation, which are dependent on the environment and are specific to cells. Recent research has been done considering 3D cell culture leading to new methods for drug transport. In the future, mathematical models will continue to help as a guiding path for understanding the tumor architecture and its growth and studying the transport of oxygen and nutrients among different zones within the spheroid via cellular and chemical dynamics.

### 3.6 Self-Assembly and Self-Sorting

**FIGURE 6:**
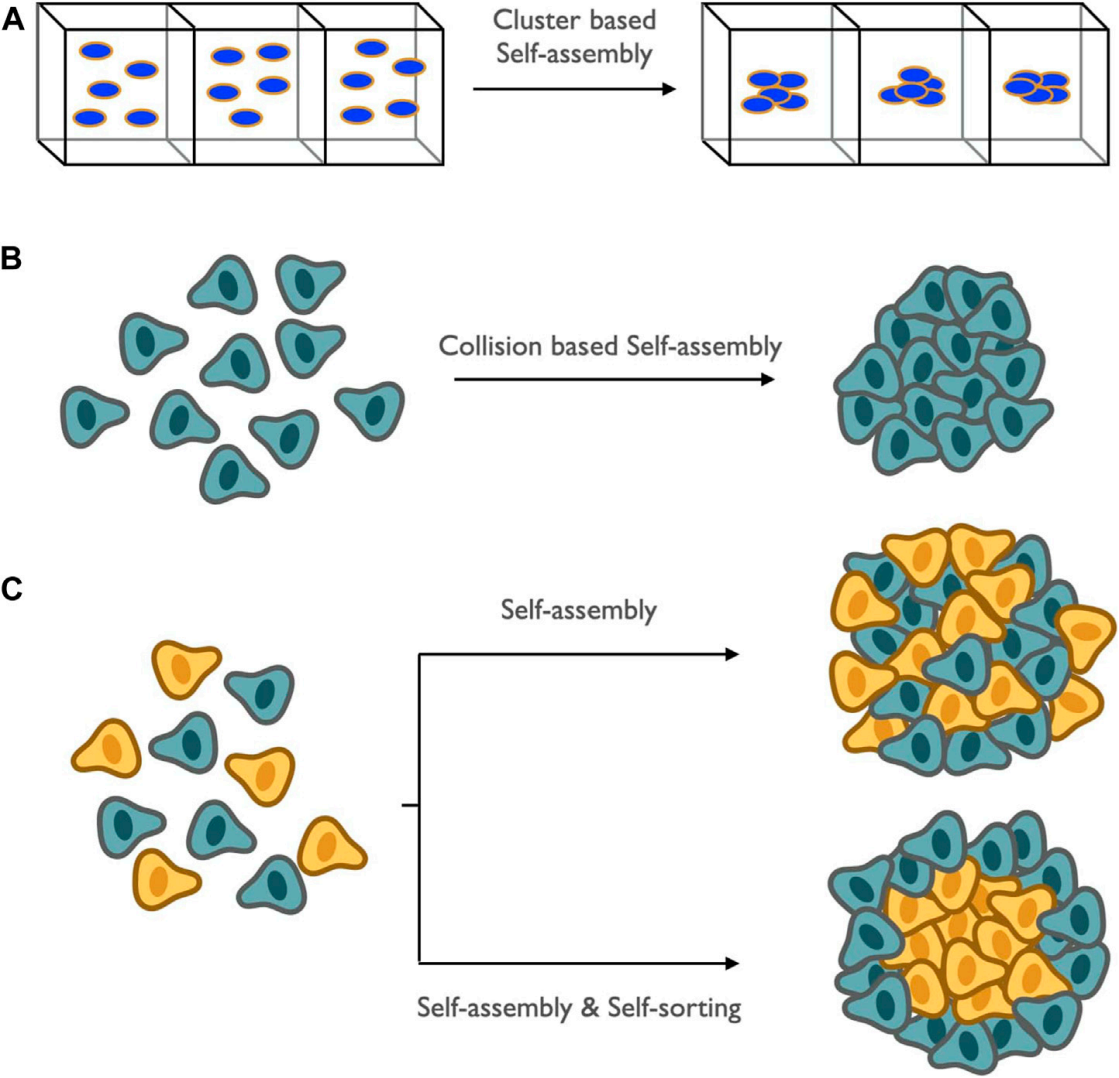
Schematic representation of spatial arrangement and assembly of proliferating and necrotic cells: it is mediated by the soluble products among themselves and regulated by convection and diffusion and is termed as self-assembly. Two broad categories of self-assembly: **(A)** cluster-based self-assembly, which involves the formation of small compartments of mono-dispersed cells; **(B)** collision-based self-assembly, which involves the arrangements or grouping which occurs when suspended cells collide among themselves. Self-assembly is always followed by sorting of mixed cell populations leading to a particular pattern of the cellular combination known as self-sorting.

The organization of interior layered composition of MCTS can be determined by the spatial arrangements, interaction, and grouping of cells combined into developmental and functional patterns. The fabrication of these similar or different cellular patterns allows them to form self-organized individual compartments assembled together, resulting in a highly stratified structure is known as self-assembly. This bio-fabricated spheroid assembly is mediated by the molecular gradients of soluble factors which are capable of binding to cellular receptors causing the signals to initiate proliferation. These soluble factors within the spheroid microenvironments are established by the process of convection and diffusion. This arrangement across the multiple length scales can be assembled in two different ways: cluster-based self-assembly and collision-based self-assembly. Cluster-based self-assembly consists of the partitioning of mono-dispersed cells into sectors followed by their settlement and aggregation, maintaining their cellular integrity as clusters and resulting in spheroid. In contrast, when the suspended cells strike into each other, leading to the formation of a spheroid is termed collision-based arrangement. Self-assembly is often followed by self-sorting. When varying cell types are organized among themselves, leading to a particular pattern of segregation, this can be stated as self-sorting (Figure 6). Based on this, a theoretical model has been developed for the study of spheroid formation that occurs when two cell types are differently segregated. Modeling of a spheroid based on self-assembly and sorting is affiliated with cell-to-cell adhesion and surface tension. This necessitates the sorting of cells of the highest cohesion to the interior of the spheroid and those with lower cohesion to the outer. This hypothesis is known as Differential Adhesion. From the recent study (Achilli et al., 2012), it has been shown that modeling of self-sorting processes can be done using an order parameter to describe the relationship between heterotypic interface length and size of the system, taking the geometrically driven argument as its base.

The subsequent part of the study will elucidate another key component of the targeted drug delivery mechanism, that is, the study of drug molecules and how they can be modeled mathematically and the key attributes related to their physical properties and chemical composition.

## 4 Kinetics of Nanomedicine in Tumor Spheroids

Anticancer drugs used for chemotherapeutic purposes to treat intractable cancers have undesired cytotoxic side effects for normal tissues due to lack of selectivity to the target diseased tissue and broad biodistribution once administered into the systemic circulation. Additionally, free drug candidates are characterized by insufficient pharmacokinetics and early degradation in the physiological environment (Duncan and Gaspar, 2011; Curtis et al., 2016; Cabral et al., 2018; Tchoryk et al., 2019). Usage of drug formulations for treating cancer having sizes greater than preferred nanometers has adverse effects such as *in vivo* instability, poor bioavailability, issues with target-specific delivery, and toxic effects. To overcome these critical medical challenges, some potential and advanced technology is needed. Nanotechnology can be used as a gateway to bridge the gap between biological phenomena and physical mechanisms. It entails the use of nanoscale materials having sizes 10–100 nm with the concept of aiming peculiar drugs to the desired cells, tissues, and body parts. To this end, drug delivery in a targeted fashion to the diseased sites is a smart strategy to combat enhanced therapeutic benefits and limit these cytotoxic side effects of drugs to normal tissue. Desired drug individuals are specially designed as nanomedicine (i.e., nano-drug formulations) so that effective therapeutic concentration of active drug molecules reaches its site of action to exhibit required pharmacological activities (Cai et al., 2019). Nanomedicine-based approaches could be used as a translational technology where drugs interact particularly with target-specific diseased tissue and individual cells with normal sites that remain thoroughly unaffected and thus ensure to mitigate undesired toxic side effects. It exerts remedial agents at the nanoscale with size ranges between 10 and 100 nm leading to the frontiers of nanomedicine drug delivery, more precisely with a controlled release (Prokop and Davidson, 2008; Jain and Stylianopoulos, 2010; Bae and Park, 2011; Carolyn and Charles, 2012; Babu et al., 2014; Marchal et al., 2015; Li et al., 2017). These remedial agents have to follow certain fundamental objectives for effective delivery mechanisms. The foremost objective of nanomedicine is to increase the concentration and the augmentative exposure of the therapeutic drug candidates to the core. Therefore, target-specific drug delivery is a very promising strategy for therapy against intractable cancer. Thus, nanomedicine ensures enhanced therapeutic efficacy and simultaneously reduces the event of systemic toxicity of anticancer drugs. But, the efficacy of nanomedicine depends on the spatiotemporal concentration distribution of the therapeutic drug candidates in the entire tumor, from the proliferation zone up to the core, which is associated with the tumor microenvironment and physicochemical properties of nanomedicine (Shyh Dar and Leaf, 2008; Markman et al., 2013; Wicki et al., 2015; Maity and Stepensky, 2016). The shape, size, charge, initial molar concentration, pH, chemical composition, the effect of targeting ligand, and cross-linking of the nanomedicines have a profound impact on its ability to diffuse, penetrate and accumulate into the solid tumor as well as tumor spheroids.

Nanomedicines are characterized by a stable circulation in the bloodstream, escape from unnecessary unspecific interactions with various blood components, successfully extravasate from blood vessel to diseased site and increase the ability of interactions and recognitions by target-tumor tissue and deliver drugs into the intracellular system. Thus, nanomedicine formulations avoid leakage and degradation of drugs in the blood compartment. A very stable blood circulation of nanomedicine is recognized by solid tumors for developing tumor-targeted drug therapy strategies. Furthermore, the tumor vasculature is leaky and non-restrictive, which offers nanomedicines an enhanced permeability to the tumor site. Once nanomedicine enters, it remains there for a long time due to impaired lymphatic drainage system (Shyh Dar and Leaf, 2008; Markman et al., 2013; Wicki et al., 2015; Maity and Stepensky, 2016b). This is enhanced permeability and retention effect (EPR), which is an outstanding mechanism for drug accumulation into tumor sites. Nanomedicine is transported through the tumor blood vessel, which is found across interendothelial gaps and follows transendothelial pathways. In addition, they have fenestration and vesicular vacuolar organelles with 50–100 nm size, which is simplified to the transport of a tiny shape nanomedicine into a tumor. Nanomedicine with 100 nm size extravasate by vascular bursts in the tumor. This process is done by intratumoral and vascular pressure gradients. It helps to ingest the nanomedicine into the tumor interstate.

Nanomedicines are internalized to target cells via the endocytosis mechanism and pass through endosomal-lysosomal vesicles. The acidic pH of the endo-lysosomal compartment acts as a trigger for some nanomedicine to release cargo drugs inside target cells and is suitable for its action. For this purpose, nanomedicine is prepared with biocompatible polymers such as PEG (polyethylene glycol), which shields the outer surface, avert elimination by RES from the bloodstream, and also extend the lifetime of nanomedicine in the bloodstream and foster further extravasation and tumor recognition processes (Li and Huang, 2009; Nie, 2010). The size and charge of nanomedicine affect the whole process. Nanomedicine size less than 150 nm accumulates in the liver and larger than 150 nm stay in the spleen. Nanomedicine with a positive charge is mostly found in the liver, spleen, and lungs, whereas neutral or negative charge nanomedicine tends to stay in the bloodstream for a longer time. A ligand-installed on the nanomedicine periphery establishes a recognition towards vascular receptors, promoting the extravasation of nanomedicines from the bloodstream into tumors (Li and Huang, 2009; Nie, 2010). Numerous nanomedicine formulations like dendrimer, liposome, drug-polymer conjugates, nanoparticles, and polymeric micelles act as tumor-targeted drug delivery vehicles (Yang et al., 2015). The design of biocompatible polymers abides by the guidelines of the FDA for biomedical applications of the polymeric micelle, where a minimum amount of polymer is administered to avoid unwanted toxicity in the body and activation of immune responses (Ahmad et al., 2010; Sun et al., 2017; Mullis et al., 2019; Tan et al., 2020). Thus, a risk-free biodegradable polymer is designed and frequently used, which disintegrates into monomer once contributing its part, excreted from the body without accumulation and toxicity. As a result, these properties can provide an insight into the fundamental mechanisms of the underlying kinetics of drug delivery and help in building up the potency of nanomedicine delivery in a targeted way (Lane et al., 2015; Donahue et al., 2019).

The entire pathways taken by nanomedicines to reach the core of spheroids can be treated mathematically. Mathematical modeling can be perceived as an essential tool for quantitative analysis of impaired drug delivery. As a result, several mathematical models have been developed to study the penetration, kinetics, and biochemical effects of therapeutics. The succeeding section will bring forth the broad vision on the optimal delivery of nanomedicine to the core of the spheroid and facilitate their tailoring. Moreover, it will address how various mathematical models are capable of forecasting the effect of different physical parameters on the spatiotemporal dynamics and penetration of nanomedicines. Profuse mathematical models have been set in motion for more elaborative and meticulous designing of nanomedicine, which will be discussed below as:

### 4.1 Mathematical Modeling of Nanomedicine

**FIGURE 7:**
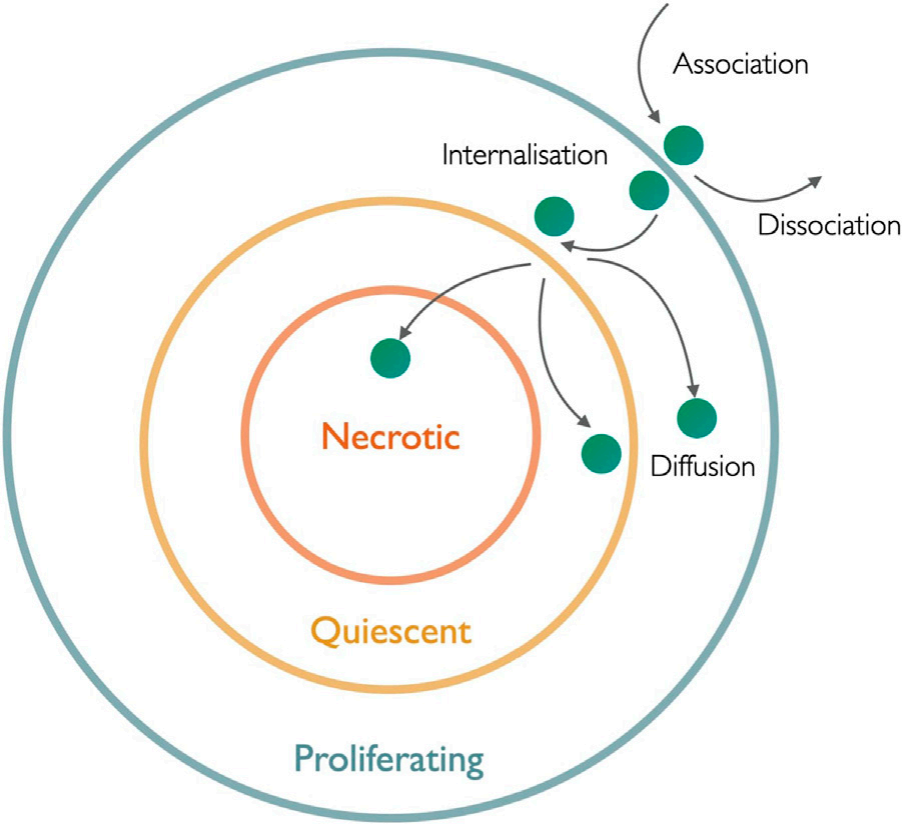
Schematic illustration of nanomedicine penetration into the hierarchical layers of the spheroid: the whole nanomedicine internalization and diffusion process depends on different rate coefficients of association, dissociation, and internalization and is depicted by arrows.

The rapidly growing nanotechnology evokes the need for understanding how nanomedicine’s characteristics influence the transport processes. Mathematical modeling has the potential to point to the comprehensive view of nanomedicine designing and characterize the important features prerequisite for drug delivery. In this section, we will discuss the quantitative insights for the elucidation of nanoparticle (i.e., nanomedicine) diffusion mechanisms in the bulk, penetration into the multicellular spheroid, and then the calculation of binding site availability on the spheroid surface (Figure 7). The viability of requisite mathematical models for the treatment of spheroids with nanomedicine will be of primary focus.

The binding of a drug molecule is essential when it comes to an unhindered drug delivery mechanism, which is proportional to the optimized value of successful binding. Molar concentration plays a major role while addressing the optimized binding and penetration of nanoparticles to and through the spheroid. Binding can be maximized in two different ways: first, it is related to the molar concentration of nanoparticles, while the other is related to the molar concentration of the cell surface binding sites in the spheroid. To study the penetration of nanomedicine to the spheroid, using stock’s solution (Gao et al., 2013), the molar concentration of nanoparticle ( $\rho^{M}$ ) in Mol/L can be calculated as follows:

$$\rho^{M}\;\left(Mol/L\right)=\frac{NP\;conc.\;\left(number/L\right)}{6\times 10^{23}particles/mole}=\frac{6C_{stock\;}\times 10^{12}}{N_{A\;}\pi \rho d^{3}},$$ (6)

where C_stock_ is the nanoparticle concentration in manufacturer stock solution, $N_{A}$ is Avogadro’s number, ⍴ is the density of nanoparticle, and d is the nanoparticle diameter in meters. The stock’s solution is being prepared for the transport study of the molar concentration and aggregation of nanoparticles. Moreover, the molar concentration of cell surface binding sites in a spheroid can be defined as follows:

$$B_{max,\;spheroid}=\frac{\phi \times 10^{15}}{6\times 10^{23}\times \frac{4}{3}\pi r_{cell}^{3}}B_{max,\;single\;cell.},$$ (7)

where ɸ is the cell density calculated by taking the volume of total cells in spheroid divided by the total spheroid volume (with the assumption of the spherical shape of spheroid), r_cell_ is the radius of the tumor cell in the suspension of stock’s solution, B_max, single cell_ is the molar concentration of single-cell binding, and B_max, spheroid_ is the molar concentration of cell surface binding site in spheroid (Gao et al., 2013).

Mathematical modeling is instrumental in aiding our understanding of the internalization of nanomedicine through the stratified structure of tumor spheroid and then followed by its diffusion inside the spheroid. The diffusion of nanomedicine at the cellular and extracellular levels is stipulated by its calculation in the interstitium and interstitial sites and taking the porosity of the spheroid into account, addressed using mathematical tools. Nanoparticle penetration to the cells can be designated as a two-step process. First, the adsorption of nanoparticles is then followed by their internalization. Fundamentally, association or this binding kinetics of negatively charged nanoparticles to cell surface termed Langmuir adsorption with the assumption that a particular cell line has a fixed capacity for particle binding through electrostatic interactions (Wilhelm et al., 2002). This process incorporates the rate of change of mass of nanoparticles bound to the cell surface, established by considering the molar concentration in the extracellular medium (M), the mass of adsorbing and desorbing nanoparticles. Langmuir adsorption can be modeled using a differential equation representing the variation of m(t) with time consisting of $k_{ass},k_{diss}\;$ as the association and dissociation rate constants:

$$\frac{dm}{dt}=k_{as\text{s}}M\left(m_{0}-m\right)-k_{diss}m,$$ (8)

where $m_{0}$ and m(t) are the maximum mass that can be bound to the cell surface initially and at a later time t, respectively. The above differential equation represents that the mass of absorbed nanoparticles per unit time is proportional to the molar concentration of nanoparticles, to the mass that is present on the cell surface that still can be adsorbed $\left(m_{0}-m\right),$ and to the mass of desorbing nanoparticles. Initially, at t = 0, m(t) = 0, the analytical description of mass of the adsorbed particles unifying both temporal dependencies and the concentration of binding nanoparticles on the cell surface can be given by

$$m\left(t,M\right)=\frac{k_{ass}M}{k_{ass\;}M+\;k_{diss}}m_{0}\left(1-e^{-\left(k_{ass}M+k_{diss}\right)t}\right).$$ (9)

The above expression can be modified to get maximum adsorbed mass with characteristic time $\;T=\frac{1}{\left(k_{ass}M+\;k_{diss\;}\right)}$ , which approximately gives. $m_{max}=\frac{k_{ass}M}{k_{ass}M+k_{diss}}m_{0}.$

After adsorption has occurred, the internalization of nanoparticles can be quantitatively approached in two ways. First, by considering the mass of the nanoparticle bound to the cell surface and another by taking the structural uniformity of the target, i.e., tumor spheroid, into account. Starting with the first approach, the global process of nanoparticle penetration into the tumor involves their binding mass to the cell surface represented at any time t by $m_{bind}\left(t\right)\;$ and their internalized mass within the cell via endocytosis represented by $m_{int}\left(t\right).$ Differential equation regulating all these phenomena can be stated as follows:

$$\frac{dm_{bind}\left(t,\;M\right)}{dt\;}=k_{ass}M\left(m_{0}-m_{bind}\right)-k_{diss}m_{bind}-\frac{dm_{int}}{dt}.$$ (10)

It has been previously assumed that the fraction of cell surface absorbs the nanoparticle, termed reactive surfaces (RS) which are regenerative and remain constant. Let $f_{int}\left(t\right),\;f_{0}$ be defined as the fraction of RS being internalized over the total available reactive surface at any time t and the maximum fraction of RS internalized by the cell, respectively. Thus, this can be represented by the differential equation with internalization rate constant $\left(k_{int}\right)$ , $\frac{df_{int}\left(t\right)}{dt}=k_{int}\left(f_{0\;}-f_{int}\right)$ . This equation can be used to calculate the rate of change of internalized mass as $\frac{dm_{int}}{dt}=\frac{df_{int}}{dt}m_{bind}$ . Using this equation, we can also calculate the total uptake of the mass of the nanoparticle $m\left(t\right)=m_{bind}+m_{int}.$

### 4.2 Influence of Spheroid Architecture on Nanomedicine Penetration and Diffusion.

Now, considering the structural uniformity, the penetration of nanomedicine into the spheroid is addressed quantitatively. Mathematical models have been developed using the nanoparticle-cell bio interface data to predict the penetration of nanomedicine into the spheroid that accounts for radial dependent changes of its internal structure. The diffusive transport and the spatial distribution of nanoparticles can be represented using nanoparticle-cell interaction parameters: association, dissociation, and internalization rate constants. The mathematical expression for the nanoparticle motion into the spheroid relationship is given by Goodman et al. (Goodman et al., 2008). The kinetics of nanoparticle internalization in 3D multicellular spheroid depends on the molar concentration of free nanoparticles available in the spheroid, bound nanoparticles, nanoparticles undergoing internalization, and concentration of available binding sites on the cell surface: M, M_b,_ M_int_, and M_bs,_ respectively. The entire ensemble of defining free, bound, and unbound nanoparticles is dependent on the force, which in turn is potentially mediated by cell-cell interaction and cell-ECM interaction. A set of partial differential equations representing the rate of change of respective concentration with time taking the structural changes of the spheroid in the radial direction and nanoparticles internalization using concentration into account is given (Graff and Wittrup, 2003; Ward and King, 2003; Hori et al., 2021; Gao et al. 2013). The molar concentration of free particles per unit time in spheroid volume is proportional to the molar concentration of unbound particles present in the accessible volume intracellularly, which are varying with radial coordinates, dissociated bound particles, and associated binding sites as well as unbound particles concentration can be written as follows:

$$\frac{\partial M}{\partial t\;\;}=\frac{D}{r^{2}}\frac{\partial }{\partial r}\left[\epsilon r^{2}\;\frac{\partial }{\partial r}\left(\frac{M}{\epsilon }\right)\right]\;+k_{diss}M_{b}-k_{ass}M_{bs}\frac{M}{\epsilon },$$ (11)

where k_ass_, k_diss_, and k_int_ are the kinetic reaction rates for nanoparticle-cell interaction corresponding to the association, dissociation, and internalization of nanoparticles in a spheroid, respectively. D is the effective diffusion coefficient, r is the radial coordinate representing nanoparticle diffusion measured from the center of the spheroid, R is the spheroid radius, and ϵ is the volumetric porosity of the spheroid, which is the ratio of the spheroid volume accessible to the particles to the total available volume. The partial differential equation elucidating the change of molar concentration of bound nanoparticles with time embedded proportionality with association rate of binding sites, dissociation, and internalization rates of bound particles can be presented as follows:

$$\frac{\partial M_{b}}{\partial t\;}=k_{ass}M{\;}_{bs}\frac{M}{\epsilon }-\left(k_{diss}+k_{int}\right)M_{b\;}.$$ (12)

The following differential equation represents the temporal variation of the molar concentration of remaining binding sites on the cell surface on which particles can still attach. It is proportional to dissociation and the internalization of bound particles and association rate of binding sites and unbound particles and also the rate of change of internalized particle related to internalization constant, respectively, as follows:

$$\frac{\partial M_{bs}}{\partial t}=\left(k_{diss}+k_{int}\right)M_{b}-k_{ass}M_{bs}\frac{M}{\epsilon },\;and\;\frac{\partial M_{i}}{\partial t}=k_{int}M_{b}.$$ (13)

The boundary conditions for the initial and at a later time t are as follows: initially, when all particles are present on the surface of the cell, the molar concentration of free, bound, and internalized particle bears no value, i.e., at t = 0, M (0,r) = M_b_ (0,r) = M_int_ (0,r) = 0 for 0 ≤r<R ; and at a later time t, the molar concentration of particles free in spheroid volume holds the same value as the molar concentration of nanoparticles outside the spheroid $\;\left(M_{0}\right)$ multiplied with porosity of spheroid, i.e., t = t, r = R, we have M(t, R) = M_0_ ϵ (R), with no radial variation of unbound particle intracellularly $\;\frac{\partial }{\partial r}\left(\frac{M}{\epsilon }\right)\left(t,0\right)=0.$ Here, M_0_ is considered to be uniform and equal to the averaged concentration of particles due to mixing in stirred vessels under the experimental conditions. Solving the above set of partial differential equations, we get the total number of particles that are retained in spheroid at time t as $\;M_{t}\left(t,r\right)=p\left(r\right)\left[M\left(t,r\right)+M_{b}\left(t,r\right)+M_{int}\left(t,r\right)\right],\;$ where p(r) is the piecewise linear window function. p(r) is used to consider the particles near the spheroid’s outer rim that need to be removed with the value of $r_{1\;\;}$ approximated to  0.95R . It acquires unity when all the particles are internalized and less than unity as per the variation of their radial position from the spheroid surface and center, i.e., p(r)=1 for $\;r<r_{1}$ ; $p\left(r\right)=\;1-\;\frac{r-r_{1}}{R-r_{1}}\;$ for $\;r_{1}<r\;\;\leq \;R$ .

Once the nanoparticles are internalized, diffusion sets are limited to the targeted delivery. Diffusion plays an indispensable role in how the nanomedicine gets transported to multilayers from cellular scale to extracellular scale. Diffusion is driven by gradients in concentration. It involves the penetration of nanomedicine to multilayers of cells prior to making it to the center. Out-turns of diffusion barriers in multilayer tissues such as cellular compaction, efflux system, and gap junctions have been solved via various models (Costa et al., 2016). The model by Gao et al. (Gao et al., 2013), for the diffusion coefficient of intercellular, porous spaces into the spheroid is represented in three steps. Initially, free diffusion $D_{0}$ nanoparticles in water at 37 °C are calculated using the Stokes–Einstein equation. This coefficient at a specified temperature is given by $D_{0}=\frac{k_{B}T}{6\pi \eta a}$ , where k_B_ is the Boltzmann constant, T is the temperature in kelvin,  η is the viscosity of water, and a is the radius of the particle. Now, this is followed by diffusion in the interstitium $\;\left(\;D_{int}\right)$ . It represents the diffusion in a porous gel matrix, which acts as an extensive connective network for cell-cell interactions and is responsible for the formation of an extracellular matrix via matrix protein. It depends on the ratio of particle radius (a) to pore radius $\left(a_{f}\right)$ and the square root of volume fraction of tumor interstitium matrix $\left(\phi_{int}\right)$ . The corresponding relation can be expressed as $D_{int}=D_{0}e^{\left(-\;\frac{a}{a_{f}}\sqrt{\phi_{int}}\right)},$ where the volume fraction of tumor interstitium matrix $\left(\phi_{int}\right)$ is defined as the ratio of the volume occupied by the particle diffused in the ECM to the total volume of all particles present before diffusion and $\phi_{int}$ > 0 always. This ratio is always less than equal to unity depending on the volume of particles that are diffused. For the calculation of $\;\phi_{int}$ , the value of collagen is used, which can be obtained by the multiplication of interstitial collagen concentration and its effective cell volume. For movements of nanoparticles to occur through the ECM, the typical mesh size of the tumor matrix should be comparable or larger than the size of the nanoparticles. Hence, the ratio $\;\frac{a}{a_{f}}\;$ is usually less than unity. Collectively, this will lead to the overall value of $(\frac{a}{a_{f}}\sqrt{\phi_{int}}$ ) less than unity. Now, incorporating diffusion at interstitial cellular scale, which mainly depends on the cellular density ϕ and porous spaces inbetween the cell (1−ϕ) can be represented as $D_{i}=D_{int}{\left(1-\phi \right)}^{2}$ ; i.e., $D_{i}=D_{int}\;\epsilon^{2},$ where D_i_ is the interstitial diffusivity constant for diffusion in a porous gel matrix with immobilized cells and ϵ is the porosity in spheroids and defined as the ratio of the volume of immobilized cells to the total volume of cells in the tumor matrix and lies in the range of 0<ϵ<1 . The interstitial diffusivity $\left(D_{i}\right)$ depends directly on the porosity factor, which acquires values less than unity, resulting in slightly lesser diffusion at the cellular scale. Thus, this entire process of diffusion can be summarized with an inequality relation: $D_{0}>D_{int}>D_{i}$ , evidently confirming the idea of the uneven rate of particle diffusion till they reach the spheroid’s core.

Taking the shape factor of spheroid into account consisting hindrances in its pores, steric hindrances due to the presence of ligand, and the tortuosity due to structural non-uniformity, the mathematical model for the nanomedicine diffusion has been given by Goodman et al. (Goodman et al., 2008). Similar to the model by Gao et al., initially, this model also takes free diffusion in the unbound medium that is not mediated by any force and potential due to cell-cell and cell-ECM interaction into consideration. The free diffusion given by the Stokes–Einstein relation can be written as $\;D_{0}=\frac{k_{B}T}{6\pi \eta a\;},$ where D_0_ is the diffusion coefficient in the unbounded medium, k_B_ is the Boltzmann constant, η is the viscosity of the liquid, and T is the absolute temperature. Now, depending on tortuosity τ(ϵ) , shape factor F, hindrances due to hydrodynamics, and the steric effects of the diffusion coefficient in the porous media L(λ), the corresponding relation for effective diffusion constant can be presented as $D\;=\;D_{0\;}\frac{L\left(\lambda \right)}{F\tau \left(\epsilon \right)}.$ Here, τ(ϵ) is related to the curvature consisting of structural aspects of the spheroid and has a profound impact on the diffusion and hydrodynamics in porous media. It is responsible for the increase in diffusion path length in the spheroid and related to the volumetric porosity (ϵ) as $\frac{1}{\tau \left(\epsilon \right)}=1-\frac{2}{3}\left(1+\epsilon \right){\left(1-\epsilon \right)}^{\frac{2}{3}}$ , where ϵ is the ratio of the volume of immobilized cells to the total volume of cells in a tumor and bears a value in the range 0<ϵ<1 . As defined, the inverse proportionality between them leads to τ(ϵ) >1. Mobility of particles in spheroid  λ is defined as the ratio of particle’s radius  a to the pore radius $r_{p}$ ; that is, $\lambda =\frac{a}{r_{p}}$ . The typical size of pore radius is comparable or greater than the particle size as $a<r_{p}$ ; therefore, λ<1 . The ideal situation of free diffusion is often affected by hindrances in the intercellular medium and tumor spheroid structure. Steric hindrances are defined as the obstructions in the path of a particle due to the presence of surrounding particles and ligands, often slowing down its motion and may stagnate the diffusion in the pore. This steric reduction along with the effects of hydrodynamic forces can be expressed as $L\left(\lambda \right)={\left(1-\lambda \right)}^{2}\left(1-2.1004\lambda \;+\;2.089\lambda^{3}-0.948\lambda^{5}\right).$ The structure factor (F) incorporates the obstructions in the spheroid pore and holds a value F > 1. Thus, the combined effect of all these defines the demeanor of diffusion in spheroid, stating an overall lesser value of D than $D_{0\;}$ upholding the real phenomenon.

Binding sites are the receptors on the surface of cells that help the drug molecules and ligands to bind with them for better signal transduction pathways. One of the most crucial parts of targeted drug delivery is the appropriate availability of these binding sites on the surface of cells with which drug molecules can attach and eventually internalize. For impaired efficacy of drug delivery to the target, calculation of the concentration of available binding sites on the cell surface is a requisite. For the porous media, a parallel pore model was developed, which established a relationship among the molar concentrations of the available binding sites on the cell surface $\left(M_{bs}\right)$ taken initially at t = 0 with the spheroid structure, porosity, structural dimension of a particle, and pore radius as (Goodman et al., 2008) $\;M_{bs}\left(0,r\right)=\frac{2}{\pi }\frac{k_{\beta }\beta \epsilon }{a^{2}r_{p}N_{A}},$ where a is the radius of the particle, r_p_ is the pore radius, N_A_ is the Avogadro’s number, and the density of remaining binding sites present on the cell surface is represented by β , whereas k_ꞵ_ is related to the variation in binding cell density on the monolayer cell culture surface to the cells in spheroid.

The surface of the tumor is well decorated with binding sites as receptors for the targeting ligand. Once the drug molecules bind to its surface, the next concern is about the calculation of the total number of cohesive particles that are bound and internalized. A model with several assumptions for a single cell considering bound and internalized particles to determine the number of cohesive particles, rate constants, and the number of binding sites is used. The concentration of particles surrounding the cell and the mean concentration of particles in the vessel are equal. A cell’s surface area is not inhibited by any other cells. Due to the continuous turnover of the cell membrane, the cell regenerates potential binding sites as the particle internalizes, as a result of which binding sites are taken to be constant, and any exocytosis will end up in the reduced value of k_int_ (Goodman et al., 2008). The model provides the relations representing the variation of number of bound particles with time proportional to the decreasing rate of dissociation and internalized particle and the positive influence of associated binding site on the cell surface along with total particle outside the spheroid as follows:

$$\frac{dN_{b}}{dt}=-\left(k_{diss}+k_{int}\right)N_{b}+k_{ass}M_{0}N_{bs}.$$ (14)

The remaining number binding site N_bs_ on the cell surface is the difference between the number of available binding sites S, which were present initially, and the number of bound particles on the cell surface N_b_:

$$N_{bs}=\;S-N_{b}\;,\;and\;\;\frac{dN_{int}}{dt}=k_{int}N_{b\;},$$ (15)

where N_int_ is the number of particles that are internalized and proportional to the internalization rate constant of bound particles. The initial number of binding sites (S) can be related to the effective segment of cell surface area that is available for binding (β) as $\beta =\frac{Sa^{2}}{4R_{c}^{2}},$ where $\;R_{c}$ is the individual cell’s radius, and a is the particle radius. On the other hand, the growth of the number of bound particles and the total number of adhered particles can be given by solving Eq. 15. Thus, we get the number of bound particles ( $N_{b}$ ) and the total number of adhered particles $\left(N_{t\;}\right)$ :

$$N_{b}=\alpha \left(1-e^{-\gamma t}\right),$$

$$N_{t}\left(t\right)=N_{b}\left(t\right)+N_{int}\left(t\right)=\alpha \left[k_{int}t\;+\frac{\gamma -k_{int}}{\gamma }\left(1-e^{-\gamma t}\right)\right],$$ (16)

where α and γ are defined as follows:

$$\alpha =\frac{k_{ass}M_{0}S}{\gamma },\;and\;\;\gamma =k_{ass}M_{0\;}+k_{diss}+k_{int\;}.$$ (17)

Here, as γ is the sum of all the rate constants, $\;\gamma >k_{ass}$ and $\gamma >k_{int}$ individually. Also, S and $M_{0}\;$ are definite quantities and greater than unity. Hence, α<1 , which leads to the fact that $N_{b}\;and\;N_{t\;}$ are less than unity and therefore sustaining our aim of greater internalization of particles into the spheroid. Solving Eq. 16 and using experimental data, the value of particle binding to the cell, rate constants, and the number of binding sites can be calculated. Thus, the mathematical models provide us the tool and allow us to calculate all the parameters such as concentration of nanoparticles, binding sites, diffusion coefficient at distinct scales, number of adhered particles, rate coefficients, and number of binding sites which are needed for successful targeted drug delivery. This quantitative approach to the problem makes us well equipped to deal with the system qualitatively as well by studying the physicochemical properties of drug carriers.

### 4.3 Role of Physiochemical Properties of Nanoparticles.

Quantitative analysis of nanomedicine in all the physical and mathematical regimes leads the way for the study of physicochemical features of the drug. The study of these properties of nanomedicine can help in establishing a suitable platform for target-oriented delivery of drugs with more adaptability (Liu et al., 2012). The following section will illustrate the key features of nanomedicines aiming at providing efficient drug delivery mechanisms, attaining the objectives, and enhancing bioactivity (Table 3).

**TABLE 3:** Attributes for nanomedicine modeling considering two important objectives, extent of penetration to the core, and expense of release of drug to the core.

Modeling parameter	Range	Limitations	Remarks
Size	The domain of 20–70 nm preferred	The larger size can be used with growth inhibitors	The smaller the size, the better the penetration
Charge	Cation and anion, due to interaction with cells, both resist the penetration up to some range, whereas neutral nanoparticles show higher penetration	Charges will affect the size of nanomedicine by forming aggregates	Owning charge leads to accumulation in the outer region and retard the diffusion
Shape	Nanocylinders and nanorods with a large aspect ratio of height/diameter of their respective sizes are preferred	Variation of shape change with other parameters like size and length is needed to be further explored	Preferential penetration depends on the surface area in contact and hence the shape
pH	Once internalized, pH-sensitive nanomedicine leads to remarkable penetration up to the core	pH-insensitive nanomedicine lowers the penetration	More pH sensitivity leads to better penetration up to the core
Chemical Composition	Biodegradable nanomedicine is preferred	Degradability	Non-biodegradable with higher concentrations will induce growth inhibition comparable to biodegradable
Cross-Linking	Crosslinked nanomedicine over uncrosslinked leads to better penetration and lower cytotoxicity	Disassembly	Cross-linking is preferred due to compact structure though sometimes disassembly leads to a better release of drugs
Ligand	Expressing nanomedicine with the ligand is helpful in selective cell targeting	Limits the diffusion sometimes due to the size difference of pores in the tumor	Endowing nanomedicine with ligands leads to deeper penetration, higher inhibition of growth but sometimes limits the transport

#### 4.3.1 Role of Size

The efficacy of nanomedicine largely depends on the distribution of the therapeutic concentration throughout the entire tumor, from the surface to center (i.e., proliferation zone to the necrotic core), so for the study of penetration profile of nanomedicine to the necrotic region, a key attribute that should be taken into account is the size of nanomedicine. In an ideal situation, the size of nanomedicine varies inversely with a diffusive capacity (Lazzari et al., 2017). Thus, smaller nanomedicines show better penetration in the spheroid. Treating nanomedicine with collagenase has a crucial impact on its penetration. Goodman et al., 2008 (Goodman et al., 2008; Cabral et al., 2011) have shown the effect of collagenase treatment on the penetration of particles. Nanomedicines lying in the diameter range of 20–40 nm accumulated to the interior of the spheroid show a dramatic increase in penetration upon treatment with collagenase, while those having a diameter of 100 nm experience restricted penetration after collagenase treatment. Moving further to the range of 200 nm, there is no penetration at all into the spheroid interior, even with collagenase treatment. Now, taking the accumulation into consideration and how the size of nanomedicine gets affected by it, it has been reported by Horacio et al. that with the size ranging from 30 to 100 nm, the accumulation of 30 nm nanoparticles was two times higher than that of 50 nm and four times more than that of 70 and 100 nm (Horacio et al., 2011). Moreover, 100 nm nanomedicine shows higher accumulation in the liver than any other organ, highlighting the significance of nanomedicine distribution to specific organs.

#### 4.3.2 Role of Charge

The surface charge on nanomedicines also affects the efficiency of the drug delivery system and their penetration. The nature of the charge not only leads to the accumulation of nanomedicines in the spheroid but also affects the rate of penetration. The neutral charge exhibits the most rapid penetration rate in contrast with the positive and negative charges. Having any kind of charge, whether cationic and anionic, manifests slower penetration due to interaction between the charged particle with the cell surface of the spheroid, resulting in deceleration of their diffusion (Gao et al., 2013; Wu et al., 2020). The interaction of nanomedicines with the cell component expelled in the extracellular medium, which is already negatively charged, leads to the change in the size of charged nanomedicines remarkably as compared to the neutral drug molecules. In support of this, a confocal microscopy-based study showed that the charged particles formed aggregates over time (Gao et al., 2013; Lazzari et al., 2017). Hence, the cationic nanomedicines lead to a high accumulation in the outer layer of the spheroid and simple surface adsorption. On the other hand, the cationic drug molecules allow a more uniform distribution in the spheroid, resulting in loss of ability to work efficiently and destruction of the inner compact spheroid structure.

#### 4.3.3 Role of Shape

The shape acts as a key component that influences the rate of tumor accumulation and therapeutic efficacy. The influence of shape on the penetration of nanomedicine is still under study. Shape affects the nanoparticle’s affinity to bind with the target. Nanoparticle’s aspect ratio, which is defined by the height vs. diameter ratio, may establish varying rates and patterns of extravasation for different tumors. The underlying role of shape on the penetration and size of nanoparticles go hand in hand. Studies by Stenzel et al. showed that the capacity of penetration is correlated to size. Thus, the shortest one displayed the highest capacity of penetration for MCF-7 cells. Numerous shapes of nanomedicine with varying sizes can be discerned. Disc-shaped nano cylinders and cuboidal nanorods of two different sizes interpreted in terms of low and high aspect ratios showed different penetration. It has been observed that nanocylinders and nano-cuboids of a higher aspect ratio represent more nanoparticle penetration inside the spheroid when plotting the normalized intensity against their normalized distance from the center is done. This comparison can be drawn more clearly by the two-photon microscopy highlighting the more accumulation of nanoparticles inside the spheroid for the higher aspect ratios of the nanocylinders and nano-cuboids sized drug molecules (Horacio et al., 2011; Lazzari et al., 2017; Costa et al., 2021). The higher aspect ratio, hence the larger surface area in contact, can be stated as one of the reasons for this favored penetration, which promotes a higher avidity between the cells and diffusion across spheroids.

#### 4.3.4 Role of pH

The pH plays a very crucial role in the modeling of nanomedicine. While anticipating the tendency of penetration of nanoparticles, pH-sensitive nanoparticles take the lead over the rest. Targeting the tumor with nanomedicine involves the diffusion of nanoparticles and their controlled release up to the core. Thus, once internalization is done, the disassembly of nanomedicine to release the drug is the point of prime focus. As we move from the proliferation zone to the necrotic zone, the region becomes more acidic due to a drop in pH in the tumor extracellular space. Immediately after internalization, this redox and acidic environment proved beneficial for the sustained release of drugs that are pH-sensitive and can penetrate deeply and more uniformly into the spheroid mass (Lazzari et al., 2017). Nonetheless, the pH-insensitive nanoparticles manifest the reduced penetration yielding to inefficient drug delivery.

#### 4.3.5 Role of Chemical Composition

Followed by pH, another pivotal aspect in the direction of better penetration is how nanomedicine is designed chemically. Lower concentration, minimum cytotoxicity, and biodegradability are the main criteria for nanomedicine and its efficient use as a drug delivery system. Additionally, the degradability of a polymer used for nanomedicine formulation has a profound impact on the cytotoxicity and growth inhibition of cells. Biodegradable and non-biodegradable polymers prepared by self-assembly behave differently for nanomedicine-mediated targeted drug delivery (Lazzari et al., 2017). Non-biodegradable nanoparticles, such as bovine serum albumin conjugated with polymethyl methacrylate, show higher penetration and deeper accumulation in the tumor via repeated mediated endocytosis and exocytosis processes, but this higher rate of penetration will provide resistance to the sufficient intercellular release of drugs leading to comparatively lower cytotoxicity, as comparable to biodegradable nanoparticles such as bovine serum albumin conjugated with polycaprolactone. When nanoparticles are conjugated chemically with these biodegradable agents, their degradability is assured, in contrast to their higher intracellular drug concentration (Lazzari et al., 2017). These non-biodegradable nanoparticles, when taken with five times higher concentration, will induce growth inhibition comparable to bio-degradable.

#### 4.3.6 Role of Cross-Linking

While considering the optimal attributes of nanomedicine, directing its penetration to the core, the idea of cross-linking emerges as a prominent one. Nanomedicine penetration, drug release, and cytotoxicity can be affected by cross-linking of the drug-loaded micelle (Lazzari et al., 2017). A crosslinked micelle is capable of moving via a transcellular pathway leading to greater cytotoxicity than the non-crosslinked or free counterpart, which got disassembled after penetration through the outer layer. Inefficacy of free drugs can be followed by their limited diffusion. In contrast, later on, it was observed by Lu et al. (Lu et al., 2014) that the compact structure of crosslinked micelle leads to the deepest penetration, but it displays the lowest cytotoxicity by creating hindrances for its diffusion to the core, thus slowing down disassembly and release of loaded drugs. A non-crosslinked micelle can degrade on the way to penetrate at the core of tumor spheroids, and as a consequence, the loaded anticancer drugs could be released somewhere, which may lead to a complex situation of penetration of free drug vs. integrated micelle.

#### 4.3.7 Role of Targeting Ligand

Surface decoration of nanoparticles with specific targeting ligands is beneficial for selectively targeting the tumor site via receptor-ligand, transporter-ligand interaction. Nanoparticles modified with various targeting ligands are used as a plan of action for selective cancer cell targeting (Lazzari et al., 2017; Nagesetti et al., 2021). The ligand-mediated delivery of nanoparticles promoted by selective cell targeting and at the same time paved the way for limited accumulation in the surrounding normal tissue leading to maximizing the efficiency of drug effects to diseased sites and minimizing the toxic side effects related to anticancer drugs. Selective tumor cell targeting with ligands decorated on nanomedicine surface, some of them can be outlined here. Nanoparticles decorated with transferrin piloted the inhibition of cell proliferation, drug penetration, and resulting regression of spheroid volume but limited the drug penetration to a certain depth only, not to the core (Lazzari et al., 2017). Nanoparticles expressed with the folic acid ligand resulted in the highest inhibition of tumor growth when analyzed in the tumor spheroid model (Lazzari et al., 2017). Different carbohydrate decorated nanomedicines resulted in the efficient delivery of the drug and noteworthy regression of tumor growth. Moreover, an aptamer-modified nanomedicine leads to a reduction in spheroid volume up to five times more comparable with non-functionalized nanomedicine by higher penetration to the core. Hence, ligand-decorated nanomedicine showed intense signaling from the periphery towards the center of the spheroid, validating the better penetration ability to the core, higher capacity of inhibition of spheroid volume growth, and significant tumor regression. However, the binding of ligands strongly to cell surface receptors and transporter sometimes limits the nanomedicine transport. The larger nanoparticles with a size range of <10 nm and 100–200 nm, i.e., between capillary pore size in normal tissue and the pore size in the tumor vasculature, respectively, provide the passive tumor targeting but also retard the transport (Gao et al., 2013). Specific ligands are decorated to nanomedicine surfaces using different chemical approaches, e.g., click chemistry, maleimide-thiol coupling, and carbodiimide coupling (Maity and Stepensky, 2016; Maity and Stepensky, 2016).

## 5 Conclusion

In order to elicit a given curative response for different intractable cancers, the effective therapeutic concentration of anticancer drug candidates should reach the site of action to conjure therapeutic benefits. However, several inexorable barriers, including untoward pharmacokinetics, lack of selectivity, degradation of drugs in harsh *in vivo* environments, and drug leaching and widespread biodistribution, act as key factors that limit inadequate drug effects to diseased sites and cause toxicity to normal tissues. However, the use of nanomedicines for tumor-targeted drug delivery overcomes the spatiotemporal distribution of drugs and avoids the side effects. Desired drug individuals are specially designed into nanomedicine to exhibit the required pharmacological activities. Nanomedicine-based approaches could be used as a translational technology where drugs interact particularly with target-specific diseased tissue and individual cells with normal sites remain thoroughly unaffected. The preclinical evaluation of the therapeutic potential of nanomedicines demands relevant models which could exactly mimic the solid tumors in the body. However, very recently, the physiological relevance and advantages of 3D tumor spheroid models and drug screening have been widely acknowledged. The conventional 2D cultures are incapable of imitating the heterogeneity and complexity of solid tumors as *in vivo* tumors grow in 3D confirmation with a specific architecture that cannot be reproduced by a 2D monolayer cellular model system. 3D tumor spheroid models possess several characteristics of real tumors, such as cell-cell interactions, cellular microenvironments (e.g., hypoxia), drug penetration, reaction and resistance, and ECM production/deposition. The tumor spheroid model bridges the gap between the 2D monolayer cultures and *in vivo* tumor tissue models. The model allows replicating the architecture of solid tumors and better investigates the pathobiology of human cancer. The potential of the spheroids model is reported to be crucial for the development of new anticancer strategies or better measures of cancer treatment. The cellular organization within the tumor spheroids is the key aspect governing the therapeutic efficacy of anticancer drugs**.** The proliferation of cells in the external layer of the spheroid causes higher consumption of oxygen. Moreover, the oxygen and nutrient gradients are reduced towards the center of the spheroid. The cell signaling pathway and the physiological communications established between cells in close contact within the spheroids makes it possible to replicate the fundamental aspects of real tumor and its microenvironments, including the proliferative rates of different cells, specific gene expressions, ECM deposition, ECM-cell, and cell-cell physical interactions, and drug resistance. Analogous to the solid tumors, the tumor spheroids display an internal layered cellular distribution, which is a result of mass transport limitations. It impedes the diffusion of nutrients, oxygen, and metabolic wastes through the tumor spheroids and creates distinct gradients. Due to the constant availability of oxygen and nutrients, highly proliferating cells form the external layers of the tumor spheroid, which is similar to solid tumors *in vivo*. Due to depletion in oxygen and nutrients, the proliferation rate decreases, and the cell metabolism decreases progressively, giving rise to the quiescent viable zone. Further decrease in oxygen, nutrient shortage, and accumulation of metabolic wastes results in cell necrosis and forms the core of tumor spheroids. The cellular organization and presence of gradients help the internal cells to exhibit specific metabolic adaptations responsible for the impaired therapeutic efficacy of anti-cancer drugs. The microenvironments act as regulating factors that govern the rate of proliferation, differentiation, and tumor progression. It imitates the physical barriers found in solid tumors, which impedes the free penetration of drug-loaded nanocarriers. The physiology and polarity of the cell signaling pathways and their gene expressions closely resemble the real tumors. These characteristics make the tumor spheroids suitable for tumor models and can be used for evaluation of drugs in the field of oncology and are well acclimated for high-throughput drug screening. Mathematical modeling of drug delivery systems is a prerequisite for effective troubleshooting during production and efficient improvement of the safety of the pharmacological treatment procedures. It provides a quantitative understanding of the underlying physical principles and profound insight into the biochemical phenomena in the drug delivery procedure. A quantitative approach to the system helps in consolidating the entire phenomenon of drug delivery as an efficient model by comprehensively characterizing the tumor growth, features assimilating the concentration gradients of various factors, tailoring nanomedicine, and the pathway taken by drug molecules. These approaches serve as invaluable tools for designing not only the tumor architecture but also the optimization of the process of diffusion through its different layers. These approaches have a contributory impact on the mathematical understanding of the wide spectrum of drug molecules administration routes starting from their adsorption on the surface till their internalization to the core. Mathematical modeling addresses the elementary components requisite for nanomedicine delivery, starting from the description of binding sites on the tumor spheroid surface to the calculation of a number of internalized particles that are sufficiently complex enough to describe the phenomenon of interest. These models describe the essential aspects of targeted drug delivery with more precision and support the experimental data and qualitative study. This mathematical vision of the problem proved significant in our better understanding of cancer biology and its treatment. It broadens the horizon of the study of nanomedicine by addressing the shortcomings of the present empirical models.

Tumor microenvironment vs. nanomedicine efficacy: despite path-breaking advancements in the modalities of cancer treatment, the mortality associated with solid tumors has not changed much in the last decade owing to the fact that various physical barriers in the tumor microenvironment limit the treatment efficacy of cancer therapeutics. Major advancements in the field of nanobiotechnology have enabled researchers to design different nanomedicines and modify their physical and chemical characteristics according to the specific tumor microenvironment. Interaction between nanomedicine and biological system at different stages of targeting to extract important factors intrinsic to the biological system, which influences the therapeutic efficacy of the targeted nanomedicine by different mechanisms including premature clearance, phagocytic engulfment by the RES system, immunological elimination, and inhibition of tumor penetration by solid tumor microenvironment complexity. However, a more clinical relevance requires a special emphasis on the analysis of structural complexity of the tumor microenvironment, which poses mechanical, chemical, biological, and hydrodynamic barriers to nanomedicine efficacy since these factors are prime for development, delivery, and screening for better therapeutic benefits.

In the pursuit of the most effective penetration, a universal shape of nanoparticles is the need of the hour. Keeping the spheroid architecture and surface properties in mind, specific shapes of nanoparticles are designed for particular spheroid model systems. However, obtaining the optimized universal shape of nanoparticles, for an effective targeted drug delivery system generically, is yet to be achieved. The different shapes of the nanomedicines, along with their size, surface properties, and parameters, should be further explored. In this regard, we need to design nanoparticles whose shape can change dynamically depending on the tumor surface, microenvironment, and internal porosity. These shape-switchable nanoparticles allow the controllable variation in their shape as per the geometrical constraints of the tumor and are able to shrink and adjust their size according to the encountered environment. This stimuli-responsive nanomedicine is promising in designing a universal nanomedicine carrier for the application in drug delivery.
